# Evidential Vulnerability of Religious Beliefs in the Context of Petitionary Prayers

**DOI:** 10.1111/cogs.70163

**Published:** 2025-12-28

**Authors:** Ze Hong, Cheneryue Zhang, Anzhuo Wang

**Affiliations:** ^1^ Department of Sociology University of Macau; ^2^ Department of Psychology University of Macau; ^3^ Independent Researcher

**Keywords:** Cognition, Religion, Belief updating, Culture

## Abstract

Petitionary prayers—requests made to a deity for specific outcomes—are widely practiced across religious traditions. While their efficacy remains a subject of theological debate, they exhibit remarkable resilience to disconfirmation. In three pre‐registered studies—a field study in China and two global surveys via Prolific—we examined how religious believers (Christians, Muslims, local deity worshippers, and Hindus) update beliefs and behaviors in response to prayer successes or failures for both hypothetical co‐religionists and themselves. Results indicate that belief updates generally follow a Bayesian pattern, with increases after prayer successes and decreases after failures, though with an asymmetry favoring belief reinforcement. Notably, participants from the Prolific sample exhibit sensitivity to the prior probability of prayed‐for events, attributing greater belief increases to improbable outcomes. Muslims predict belief increases even after failed prayers, consistent with doctrines framing hardships as divine tests. Across traditions, believers estimate continued prayer regardless of past outcomes, with monotheists displaying stronger resilience. These findings illuminate the cognitive and cultural mechanisms that buffer religious beliefs against counter‐evidence, contributing to debates on the evidential vulnerability of religious credence and its parallels with epistemically self‐sealing belief systems.

## 1 Introduction

Petitionary prayers—requests made to a deity for specific outcomes—are a central feature of many religious traditions. These prayers offer a compelling context for exploring human–god relationships, which Horton (1960) argues can often be analogized to human–human interactions. At one extreme, in many polytheistic traditions, supernatural entities may be manipulated or even coerced (Cohen, 1978; Hong, Slingerland, & Henrich, 2024). More commonly, however, they are supplicated, as humans acknowledge the superior power of these entities and seek to achieve specific ends by pleasing them, often through acts of worship and sacrifice (Pedrick, 1982). While monotheistic traditions like Christianity and Islam may theologically call into question the purpose of petitionary prayers by arguing that an omnipotent and omniscient God should have already devised the best plan for humanity (Howard‐Snyder & Howard‐Snyder, 2010; Davison, 2021), such prayers remain prevalent among believers (Brown, 1966; Halman, 2022). This prevalence spans both Western (Finney & Malony, 1985) and non‐Western contexts (Nel, 2022), with many believers claiming that God answers their prayers, often citing personal experiences as evidence (Brümmer, 2017). Belief in the efficacy of prayer underpins practices like faith healing, which is often positioned as an alternative to modern medicine (Peters, 2007). Notably, even scientifically minded researchers have attempted to test the efficacy of prayer through experimental studies (Galton, 2012; Joyce & Welldon, 1965).

An intriguing feature of petitionary prayers is their apparent resilience to empirical disconfirmation. Failures of prayer often do not diminish faith in God, particularly in monotheistic traditions (Brümmer, 2017). Instead, failed prayers are easily rationalized by invoking God's mysteriousness (Boudry & De Smedt, 2011), the insincerity of the prayer (Kling, 2020), or the petitioner's impure motives (Gellman, 1997). This resilience has fueled broader discussions in the cognitive science of religion about the nature of religious beliefs and their susceptibility to empirical evidence (Boudry & Coyne, 2016a, 2016b; Levy, 2017; Oviedo & Szocik, 2020; Van Leeuwen, 2016). This apparent invulnerability of petitionary prayers is puzzling given that they are by definition goal‐directed behaviors with outcomes that are typically observable and subject to evaluation, and one would expect empirical feedback to influence beliefs about their efficacy (Hong & Henrich, 2021). Indeed, much psychological research shows that goal‐directed behaviors are heavily influenced by outcomes within the framework of reinforcement learning (Botvinick et al., 2019; Dayan & Balleine, 2002). From the perspective of rational Bayesian belief updating, failures of prayer should not be entirely dismissible; although “auxiliary hypotheses” (e.g., lack of sincerity in prayer) may absorb much of the blame for failed outcomes, core beliefs (e.g., that God exists and is benevolent or capable) should nonetheless be affected (Gershman, 2019). If beliefs about God's willingness or power to answer prayers were completely unresponsive to evidence, this might suggest that religious beliefs are qualitatively distinct from ordinary factual beliefs, as some scholars have suggested (Van Leeuwen, Weisman, & Luhrmann, 2021). Yet, evidence indicates that religious beliefs are often affected by worldly events (Dein, 2001) and significantly influence everyday behavior, even in ways that are harmful to the believers (e.g., faith healing as a substitute for medicine) that would be hard to explain without assuming sincere factual belief (Boudry & Coyne, 2016a, 2016b; Hong & Boudry, 2025).

In the present studies, we contribute to this debate by empirically examining how participants with various religious backgrounds evaluate changes in belief and behavior in hypothetical religious protagonists when faced with successes or failures of petitionary prayers. Specifically, in three sets of pre‐registered studies (a field sample collected from various localities in China and two global samples obtained through the commercial survey platform Prolific), we asked participants with different religious affiliations (Christianity, Islam, local deity‐worship/Hinduism, and atheism) to read short vignettes of praying for specific outcomes (e.g., passing an important exam, recovering from an illness with high survival rate, recovering from an illness with low survival rate, and returning safely from long‐distance travel) in either third‐person (protagonists sharing the participants’ religious affiliation) or second‐person (participants themselves) perspectives, where participants were asked to imagine how beliefs in God/gods and future praying behaviors may change in response to prayer successes or failures. The inclusion of a non‐believer group serves both as a methodological baseline that captures predictions grounded in secular folk psychology and as a way to probe how non‐religious individuals perceive the belief dynamics of religious others.

Given that religious beliefs are, at least on the surface, resistant to falsification, yet Bayesian principles predict that they should respond to counter‐evidence, however slightly, we propose three main hypotheses applicable to all participants. First, we predict that belief change will always be rated as changing in the Bayesian rational direction (i.e., increase in case of prayer successes, decrease in case of prayer failures; H1). Second, we hypothesize that the magnitude of belief increase due to prayer success will exceed the magnitude of belief decrease resulting from prayer failure (H2). This asymmetry may arise because positive religious experiences often reinforce faith more strongly than negative experiences weaken it as a result of confirmation bias (Nickerson, 1998) and motivated reasoning (Kunda, 1990), where people tend to selectively interpret information in ways that support their existing beliefs and favor conclusions that align with their desires or commitments. Third, we predict that when the probability of the prayed‐for outcome occurring without divine intervention is low (e.g., pancreatic cancer with a 10% 5‐year survival rate), rated belief increases will be greater in the case of prayer success, and belief decreases will be smaller in the case of prayer failure, compared to situations where the probability of the prayed‐for outcome occurring without divine intervention is high (e.g., thyroid cancer with a 60% 5‐year survival rate; H3). This follows from the assumption that people are sensitive to Bayesian priors (the prior probabilities of events occurring based on available knowledge) such that they are more likely to attribute unlikely outcomes to divine intervention and adjust their beliefs accordingly.

We also have hypotheses regarding group‐level differences. For monotheistic religious believers (Christians and Muslims), we predict that the rated decrease in belief in case of prayer failure will be smaller than for local deity worshippers and non‐believers (H4). This is because both Christianity and Islam emphasize the total control of one single omnipotent, omniscient, and benevolent entity as well as the submissive attitude of believers (Schoenfeld, 1992). In these traditions, unanswered prayers are often interpreted not as evidence against God's power but as part of a larger divine plan, reinforcing the idea that human understanding is limited (McCann & Johnson, 2022). In contrast, local deity worshippers often maintain a more transactional relationship with their deities, perceiving them as powerful but not necessarily benevolent, omniscient, or even consistently capable (Hansen, 2014), in a way not very different from powerful worldly individuals that people may interact with in a secular setting (e.g., a mafia boss). If a local deity fails to grant a request, worshippers may infer that the deity is unreliable or unworthy of further devotion, leading to a greater decline in belief. Thus, while belief change should still align with Bayesian principles, we expect that prayer failures will have a smaller impact on belief reduction among Christians and Muslims because their religious frameworks provide built‐in rationalizations for unanswered prayers (a point further elaborated in the discussion section). In contrast, local deity worshippers, lacking such theological justifications, may exhibit greater belief fluctuations in response to perceived divine efficacy.

Regarding rated behavior changes, we predict that while for local deity worshippers and non‐believers they will be in the same direction as rated belief changes, for Christians and Muslims behavior changes will be rated to increase even in the case of prayer failures (H5). This is because these monotheistic traditions often instruct followers to engage in actions to strengthen their belief, especially in cases of weakened belief and doubt (Festinger, Riecken, & Schachter, 1956; Luhrmann, 2020).

Finally, we predict that participants will estimate smaller belief decreases in response to prayer failures in second‐person vignettes, compared to third‐person vignettes (H6). This is based on the expectation that individuals perceive their own faith as more resilient than that of their co‐religionists. This effect may be especially pronounced among Christians and Muslims, as monotheistic traditions like Christianity and Islam place strong emphasis on unwavering faith in an omnipotent God.

## 2 Study 1

In Study 1 (preregistered at https://osf.io/r9ezj/?view_only = a5ca10e9ce8a4ee8a2afed09e1fe5fee), we aim to investigate how participants from different religious groups in China evaluate changes in belief and behavior when faced with successes or failures of petitionary prayers in the third‐person perspective. By using vignettes tailored to each group's religious context, we explore the perceived evidential vulnerability of religious beliefs and behaviors in response to observed outcomes of prayers. The original survey questions and Qualtrics links can be found in the Supporting Information A. Ethics approval (for all three studies) was obtained from the ethics board of the University of Macau (Approval number SSHRE24‐APP001‐FSS).

### 2.1 Method

*2.1.1. Participants*

We collected data from four participant groups: Christians, Muslims, local deity worshippers, and non‐believers. Christian participants (*n* = 52, age = 29.76 ± 14.18, 39.12% male) were recruited from a major Methodist church in Fuzhou, Fujian province, and Muslim participants (*n* = 133, age = 37.48 ± 10.93, 57.89% male) were recruited from Shadian, Yunnan province—two regions with significant populations of these respective faiths. Both groups were recruited through the authors’ personal connections, as the academic study of religion is a sensitive topic in China, and believers are often reluctant to engage with unfamiliar interviewers. For the Christian sample, one co‐author was a regular attendee of the church, and her parents, who served organizational roles in the congregation, recommended some participants. For the Muslim sample, another co‐author grew up in the town where the study was conducted and, together with her parents, suggested potential participants. Although the co‐authors did not personally know all participants, these recruitment methods may have biased the samples toward individuals who were more active in their religious communities, more trusting of the co‐authors’ families, or more willing to participate in research endorsed by local religious leaders. In the case of the Muslim sample, many of the co‐author's personal contacts were highly educated young individuals who tended to be more skeptical of traditional tenets; these individuals were excluded from the final analysis.

Local deity worshippers (*n* = 71, age = 29.25 ± 14.72, 39.13% male) were recruited through convenience sampling at Guangyue Temple, Kaiyuan Temple, and Tianhou Temple in Quanzhou, which are among the most popular sites for local deity worship in southern China and have numerous sub‐temples in various cities. Non‐believers (*n* = 50, age = 20.96 ± 3.77, 37.25% male) were recruited via social media, also leveraging the authors’ personal networks.

*2.1.2. Procedures*

All participants first answered questions about their belief in God (or gods) and how often they engaged in religious activities. They then proceeded to respond to vignette scenarios by completing a survey on their mobile phones after scanning a QR code provided in person by the researchers. Table 1 lists all the vignette scenarios, which were originally designed for Christian participants. For participants in other religious groups, the vignettes were adapted to reflect their religious context by substituting the name of the supernatural deity (e.g., “Allah 真主” for Muslims and “local deities [names of specific local deities]” for local deity worshippers). To ensure that participants in our samples were sufficiently religious, we excluded responses from participants who indicated that they either “strongly disbelieve” or “somewhat disbelieve” in the existence of God or gods. This procedure resulted in final sample sizes of 44 Christians, 84 Muslims, and 44 local deity worshippers.

**Table 1:** Vignette scenarios used in Study 1

Scenario	Scenario Detail and Question
Exam [failure/success]	Belief question: [Protagonist #1] is a devout Christian who prays and worships regularly. Before taking his college entrance exam, he prayed earnestly to God to be admitted to his desired school and also studied hard for the exam. However, when the results came out, [Protagonist #1] [did not perform well and failed to get into/performed well in the exam and got into] the school of his choice. Do you think [Protagonist #1]’s belief in God will change because of this? Behavior question: After repeating a year and taking the college entrance exam again, what do you think the likelihood is that [Protagonist #1] will pray to God?/After his successful admission into his dream school, what do you think the likelihood is that [Protagonist #1] will pray to God?
Illness high survival [failure/success]	Belief question: [Protagonist #2] is a devout Christian who regularly prays and participates in worship. His mother was suddenly diagnosed with thyroid cancer (which has a 40% mortality rate). He prayed earnestly to God, asking for his mother to recover. His mother had a strong will to live and cooperated with the medical treatment [but she passed away painfully a month later/and she recovered her health a month later]. Do you think [Protagonist #2]’s belief in God will change because of this? Behavior question: After this, [Protagonist #2] himself becomes ill in his old age, and he strongly hopes to recover because he still has many worldly concerns. What do you think is the likelihood that [Protagonist #2] will pray to God?
Illness low survival [failure/success]	Belief question: [Protagonist #3] is a devout Christian (who regularly prays and participates in worship). His mother was suddenly diagnosed with pancreatic cancer (which has a 90% mortality rate). He prayed earnestly to God, hoping for his mother's recovery. His mother had a strong will to live and cooperated with the treatment [but she passed away painfully a month later/and she recovered her health a month later]. Do you think [Protagonist #3]’s belief in God will change because of this? Behavior question: After this, [Protagonist #3] himself becomes ill in his old age, and he strongly hopes to recover because he still has many worldly concerns. What do you think is the likelihood that [Protagonist #3] will pray to God?
Safe travel [failure/success]	Belief question: [Protagonist #4] is a devout Christian who prays and worships regularly. His father often has to travel for work, and before going on a long‐distance flight for a business trip, [Protagonist #4] prayed earnestly to God for his father's safe return. [The flight went smoothly, and [Protagonist #4]’s dad returned home safely after the business trip/Unfortunately, the plane was involved in an accident, and his father tragically died]. Do you think [Protagonist #4]’s belief in God will change because of this event? Behavior question: Afterward, [Protagonist #4]’s mother also needs to take a plane for a business trip due to work. What do you think is the likelihood that [Protagonist #4] will pray to God?

*Note*. The original materials were translated from Mandarin Chinese into English using ChatGPT, with all translations manually verified by the first author.

**Table 2:** *p*‐values and effect sizes for estimated belief changes in prayer failure scenarios among Christian and local deity worshippers

Group (Scenario)	Mean	SD	*t* (df)	*p*	Cohen's *d*
Christian (exam failure)	−0.386	1.166	−2.199 (43)	.017*	−0.331
Christian (illness low survival failure)	−0.273	1.420	−1.273 (43)	.105	−0.193
Christian (illness high survival failure)	−0.295	1.503	−1.304 (43)	.010**	−0.197
Christian (safe travel failure)	−0.674	1.340	−3.300 (42)	<.001***	−0.503
local deity worshipper (exam failure)	−1.412	1.327	−1.412 (40)	.083	−0.220
local deity worshipper (illness low survival failure)	−0.565	1.399	−0.565 (39)	.288	−0.089
local deity worshipper (illness high survival failure)	−1.317	1.320	−1.317 (39)	.098	−0.208
local deity worshipper (safe travel failure)	−2.411	1.377	−2.411 (39)	.010**	−0.381

*Note*. Statistical significance is indicated at the levels of .05 (*), .01 (**), and .001 (***).

The study employed a within‐subjects design, featuring four types of prayers (exam success, illness with high survival probability, illness with low survival probability, and safe travel). Each prayer type had two possible outcomes (success or failure), resulting in a total of eight sets of questions per participant. Each set consisted of one belief‐related question and one behavior‐related question. Participants rated estimated changes in belief and future prayer behavior using a 7‐point Likert‐type scale ranging from −3 to +3. For belief questions, response options included: −3 = “*definitely decrease*,” −2 = “*very likely to decrease*,” −1 = “*may decrease*,” 0 = “*remain the same*,” +1 = “*may increase*,” +2 = “*very likely to increase*,” and +3 = “definitely increase.” The same scale was used for behavior questions, referring to the likelihood of future prayer behavior. For non‐believers, the protagonists in the vignettes were presented as Christians, given that most non‐believers were familiar with Christian doctrines and practices.

For Christian, Muslim, and non‐believer participants, we also conducted brief, open‐ended interviews before presenting them with the vignette questions. For religious participants, we asked two questions about the content and form of their prayers: “What do you usually pray for?” and “How do you usually pray?” Additionally, we asked two questions regarding events that might influence belief in God: “Have you ever experienced events or situations that significantly affected your belief in God?” and “What kind of events or situations do you think could weaken a devout believer's faith or even lead to a loss of belief?” The last question was intentionally chosen to specifically explore potential “defeaters” of faith given our interest in the evidential vulnerability of religious beliefs. For non‐believers, only the last question (with a hypothetical Christian protagonist) was asked. These qualitative interviews provided additional insights into participants’ perspectives on religious belief, its stability, and the factors that might contribute to its erosion.

### 2.2 Results

**Fig. 1:**
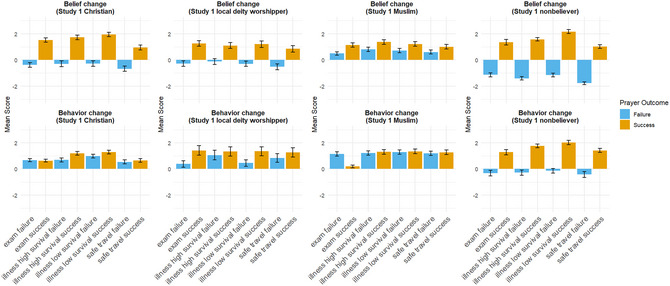
Combined results of the vignette responses from participants with different religious affiliations in our field sample in China.

The main results for our field participants from different religious denominations in China, including Christians, Muslims, local deity worshippers, and non‐believers, are summarized in Fig. 1. A complete report of all statistical tests for each hypothesis is available in Supporting Information B.

Hypothesis 1 predicted that belief in God/gods would increase following successful prayers and decrease following failed prayers. This hypothesis is partially supported. While participants across all groups estimated an increase in belief after successful prayers, belief did not consistently decrease following failed prayers. Most notably, Muslim participants estimated that belief in God would increase even when prayers fail, across all vignette scenarios (a point that will be discussed in detail later). For Christians and local deity worshippers, estimated belief changes following prayer failures are in the negative direction but do not always significantly deviate from 0 (see Table 2 for p‐values and effect sizes from one‐tailed one‐sample t‐tests against 0 for Christian and local deity worshipper participants). In contrast, non‐believers estimated large decreases in belief following prayer failures, with the magnitude roughly equivalent to the belief increase observed in the case of prayer successes.

Hypothesis 2 predicted that the absolute magnitude of belief change would be greater following prayer successes than prayer failures. This hypothesis is largely supported. With the exception of non‐believers, the magnitude of belief changes in the case of prayer successes consistently exceeds that of prayer failures. This pattern is also evident among Muslim participants, who estimated that the Muslim protagonists’ belief in God would always increase but predicted larger increases when prayers are “answered.”

Hypothesis 3 proposed that belief change would be greater for low‐probability events (e.g., recovery from pancreatic cancer) than for high‐probability events (e.g., recovery from thyroid cancer), consistent with Bayesian sensitivity to prior probabilities. This hypothesis is not supported. There are no statistically meaningful differences between participants’ responses for illness scenarios with low survival probability, compared to those with high survival probability. In other words, participants from our field samples did not seem sensitive to the natural occurrence probability of the prayed‐for events. The sole exception is observed among non‐believers: For illnesses with low survival probabilities, the estimated magnitude of belief increase following prayer successes is significantly larger than for illnesses with high survival probabilities (paired *t*‐test, *p* < .001, *t*(48) = 4.795, Cohen's *d* = 0.685). Similarly, for prayer failures, the magnitude of estimated belief decreases is smaller for illnesses with low‐probability survival, compared to high‐probability ones, though the magnitude of this difference is not as pronounced (paired *t*‐test, *p* = .032, *t*(48) = 1.900, Cohen's *d* = 0.271).

Hypothesis 4 predicted that belief decreases following prayer failures would be greater among local deity worshippers and non‐believers than among Christians and Muslims. This hypothesis is not supported. The belief change patterns of Christian participants are highly similar to those of local deity worshippers, with no discernible differences in the magnitude of belief decreases following prayer failures. Although non‐believers do exhibit larger decreases, Muslims do not show decreases at all, predicting instead small belief increases even after failed prayers.

Hypothesis 5 concerned predicted future prayer behavior, specifically that Christians and Muslims would estimate continued prayer regardless of prior outcomes, whereas local deity worshippers and non‐believers would show reduced behavioral commitment after failures. This hypothesis is partially supported. As predicted, Christian and Muslim participants consistently estimated that protagonists would continue to pray regardless of the outcome of past prayers. Contrary to expectations, however, local deity worshippers exhibit a similar response pattern, estimating high likelihoods of future prayer in both success and failure scenarios. Non‐believers are the only group for whom the estimated likelihood of future prayer following failed prayer outcomes is negative, though this difference is not always statistically significant from zero.

Our qualitative interviews with Muslim participants suggest that a substantive proportion (seven out of 15 participants who accepted and completed the interview) perceived the Muslim belief in God as absolute, viewing prayer failures as either a test or a challenge from God.^1^ One interviewee (male, 24 years old) insightfully remarked, *“For a devout Muslim, no situation can diminish or eliminate their faith. All good and bad things come from God's will, and both contain dual aspects. Ultimately, everything is an expression of God's mercy towards us. Beneath the surface of bad situations lies unseen goodness, which is also a form of divine compassion. It all depends on the perspective from which we, as humble beings, seek to understand it.”*

In contrast, some Christian participants acknowledged that prayer failures could, at times, impact believers’ faith in God. One participant (female, 50 years old) noted, *“If someone faces constant setbacks and doesn't feel like their prayers are being answered, they might start to doubt their faith. It's about the feedback they get from life.”* Indeed, 30% (14 out of 46) of the Christian interviewees explicitly mentioned that prayer failures—despite devout worship—could potentially shake a believer's faith. When including those who cited life setbacks more generally, the proportion of individuals who identified some form of empirical disappointment as a factor that might weaken belief in God rises to 67%.^2^

Interestingly, many participants who suggested that life setbacks or unanswered prayers might weaken a person's faith nonetheless maintained that their own faith remained unaffected (or even strengthened) as a result. They often rationalized their initial frustration with unanswered prayers in retrospect as part of God's greater plan. For example, one Christian interviewee (male, 17 years old) reflected, *“There were times when I prayed for things that did not happen, and initially, I felt frustrated. However, looking back, I realized that God's plans were better than my own.”*

## 3 Study 2

Though our field study sheds light on debates surrounding the evidential vulnerability of religious beliefs, Study 1 is limited by its small sample size and its recruitment from a single cultural context (China). This is a critical limitation, as the lived experience and theological emphasis of Christians and Muslims in China may differ substantially from those in other regions due to distinct cultural and political factors. To address this lack of cultural diversity and test the generalizability of our findings, we conducted a second study via the commercial survey platform Prolific, aiming to replicate our findings (preregistered at https://osf.io/9nek3/?view_only=cf49221d085c4245beaa79323b2dc016) with a larger and more diverse global sample encompassing participants from different religious affiliations. This approach allowed us to assess whether the patterns observed in Study 1 generalize across a broader population. The surveys were conducted in English and we used pre‐screening filters to select individuals who were Christians/Muslims/Hindus/atheists, fluent in English, and engaged in both public and private religious activities. No qualitative data were collected. For a detailed breakdown of participants’ demographic information, see Supporting Information A.

### 3.1 Method

#### 3.1.1 Participants

We collected data from four participant groups: Christians, Muslims, Hindus, and non‐believers (atheists). Hindus were chosen because Prolific does not have an equivalent of “local deity worshippers” as in our field sample, and Hinduism resembles Chinese folk traditions in polytheistic worship (Daniélou, 2017). Participants were screened through Prolific's pre‐screen system by specifying eligible participants’ belief as Christianity (*n* = 300, age = 32.69 ± 11.00, 37.13% male), Islam (*n* = 300, age = 30.06 ± 9.59, 64.42% male), Hinduism (*n* = 200, age = 37.80 ± 9.98, 44.16% male), and atheist/irreligious (*n* = 100, age = 32.81 ± 11.18, 69.00% male).

#### 3.1.2 Procedures

All participants took the same survey as in Study 1, with the names of the protagonists replaced by typical names in respective religious traditions (e.g., David for the Christian protagonist, Ali for the Muslim protagonist, Aditi for the Hindu protagonist), and non‐believers (atheist participants) completed the Christian vignettes as in Study 1. The English translation was performed by ChatGPT and manually verified by the first author. All participants were paid £0.7 for completing the survey, in line with the minimum payment rate specified by Prolific (£6 per hour).

### 3.2 Results

**Fig. 2:**
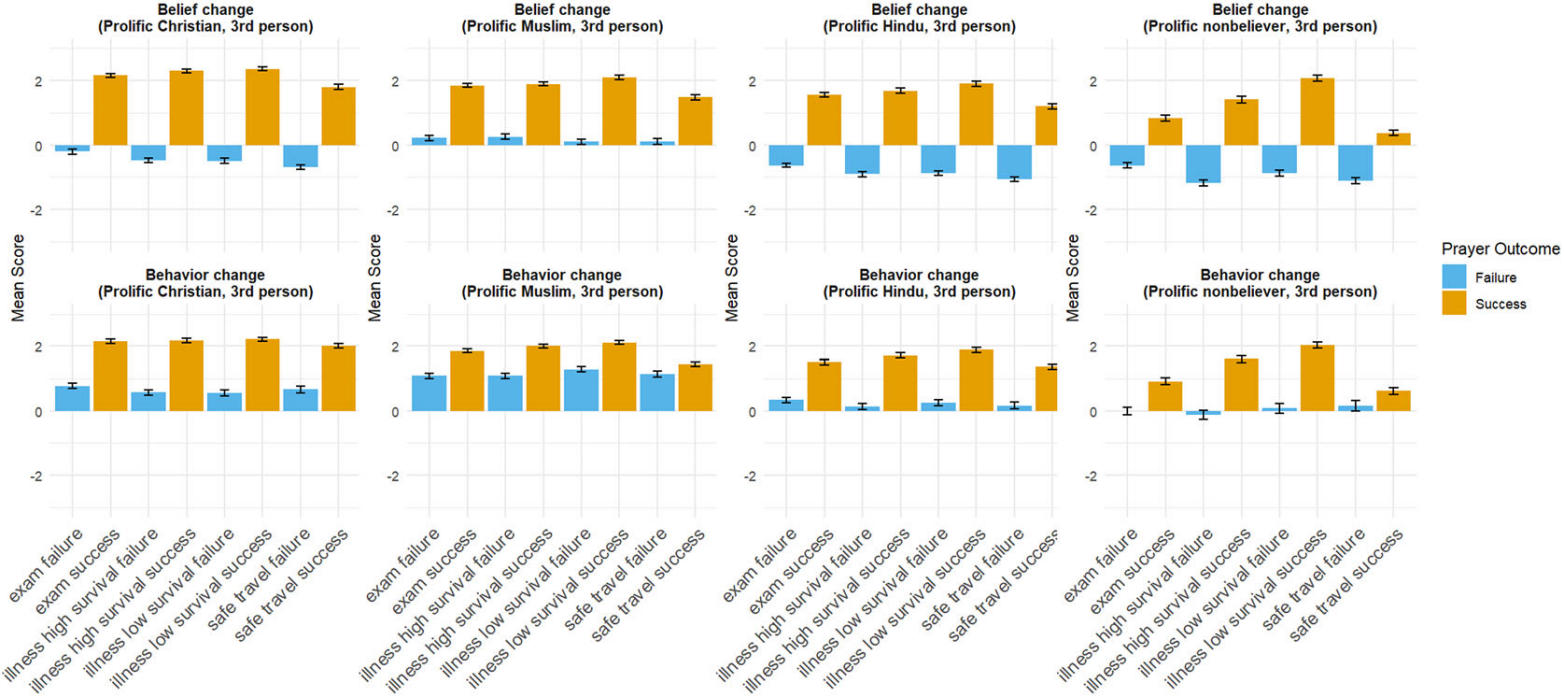
Combined results of the vignette responses from participants with different religious affiliations in a global sample recruited through Prolific.

The main results of Study 2 are shown in Fig. 2 (a full report of all statistical tests for each hypothesis is available in the Supporting Information B). Most of the patterns observed in Study 1 are replicated. With the exception of Muslims, all participants estimated the protagonists’ belief changes to follow a Bayesian rational direction, with belief increasing following prayer successes and decreasing following prayer failures. Additionally, the magnitude of belief increases in response to prayer successes is generally larger than the magnitude of belief decreases following prayer failures. Among non‐believers, the estimated decrease in belief for Christian protagonists after prayer failures is sometimes larger than the estimated increase in belief following prayer successes, as observed in Study 1. The difference in estimated belief changes suggests that non‐believers may overestimate the impact of prayer failures on Christian beliefs.

As in Study 1, Muslim participants estimated that the Muslim protagonists’ belief in God would increase even in the case of prayer failures, though the magnitude of this increase would be very small. Regarding estimated future prayer behavior, all religious participants predicted that the protagonists would be more likely to engage in future prayers, even when previous prayers had failed. However, for Hindu participants, such increases are not always statistically significant.

While most of the patterns from Study 1 are replicated, there are a few notable exceptions. Most significantly, participants in three (Muslim, Hindu, non‐believer) out of the four samples exhibit sensitivity to the prior probabilities of events occurring. That is, they estimated that protagonists’ belief in God/gods would increase more when a patient recovers from an illness with a low natural recovery probability, though the effect sizes are small. Notably, this sensitivity does not appear to apply to belief decreases in response to prayer failures, except for non‐believers (paired *t*‐test, *p* < .001, *t*(99) = 3.129, Cohen's *d* = 0.313). Another key difference between participants recruited from Prolific and those from our field sample is that belief decreases among Hindus in the Prolific sample following prayer failures are noticeably more pronounced than those observed among local deity worshippers in Study 1.

## 4 Study 3

Thus far, we have examined how religious participants estimate the belief changes and future praying behaviors of hypothetical co‐religionists in response to prayer successes and failures. However, participants may perceive their own faith as more resilient than that of their peers. To investigate this potential discrepancy, we recruited an additional sample of Prolific participants and reformulated all vignette questions in the second‐person perspective (e.g., *“Imagine your mother (or a loved one) is suddenly diagnosed with thyroid cancer…”*). This approach allowed us to assess whether participants predicted different beliefs and behavioral responses when considering their own faith rather than that of others. This study was preregistered at https://osf.io/4spvm/?view_only=864c9867076743baa32bd51a94386206. As in Study 2, the surveys were conducted in English, and we used pre‐screening filters to select individuals who were Christians/Muslims/Hindus/Atheists, fluent in English, and engaged in both public and private religious activities. No qualitative data were collected.

### 4.1 Method

#### 4.1.1 Participants

We collected data from three participant groups: Christians, Muslims and Hindus. Participants were screened through Prolific's pre‐screen system by specifying eligible participants’ belief as Christianity (*n* = 200, age = 31.88 ± 11.63, 31.25% male), Islam (*n* = 200, age = 30.64 ± 9.49, 54.67% male), and Hinduism (*n* = 200, age = 32.30 ± 11.01, 48.84% male). Note that we did not include an atheist group, as atheists by definition do not engage in petitionary prayer.

#### 4.1.2 Procedures

Participants completed the same survey as in Study 2, with all vignette scenarios reformulated from a third‐person perspective to a second‐person perspective (e.g., replacing protagonist names with “you” and making necessary grammatical adjustments). All participants were paid £0.70 for completing the survey, in line with the minimum payment rate specified by Prolific (£6 per hour).

### 4.2 Results

**Fig. 3:**
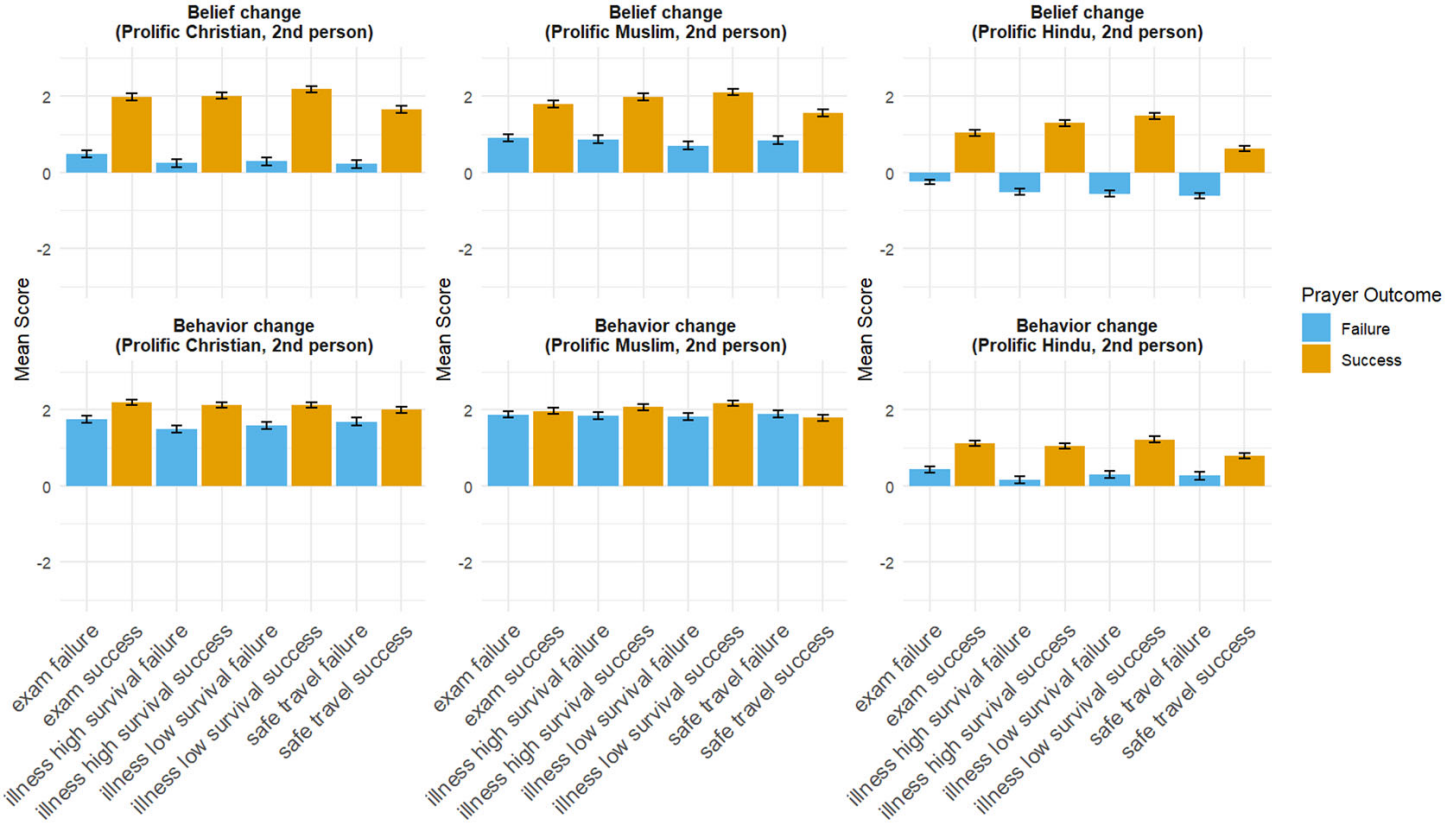
Combined results of the second‐person vignette responses from participants with different religious affiliations in a global sample recruited through Prolific.

As shown in Fig. 3, the overall patterns of belief and behavior updating remain consistent (a full report of all statistical tests for each hypothesis is available in Supporting Information B). Most notably, in the second‐person scenarios, participants once again exhibit Bayesian sensitivity regarding illness scenarios with different natural recovery probabilities—showing greater belief increases following prayer successes when the natural survival probability is low—though the effect sizes are also small (Cohen's *d* < 0.2 for all three scenarios). No statistically significant difference in belief change is observed for prayer failure scenarios.

Nonetheless, several responses in the second‐person scenarios diverge meaningfully from those in the third‐person cases. In particular, Christian participants exhibit a response pattern more similar to that of Muslims, predicting a (small) increase in their own belief in God even following prayer failures. Among Muslim participants, the magnitude of belief increase in response to prayer failures is greater than in the third‐person scenarios. Hindu participants, while still anticipating a belief decrease following prayer failures, reported a smaller reduction in magnitude compared to the third‐person scenarios.

**Fig. 4:**
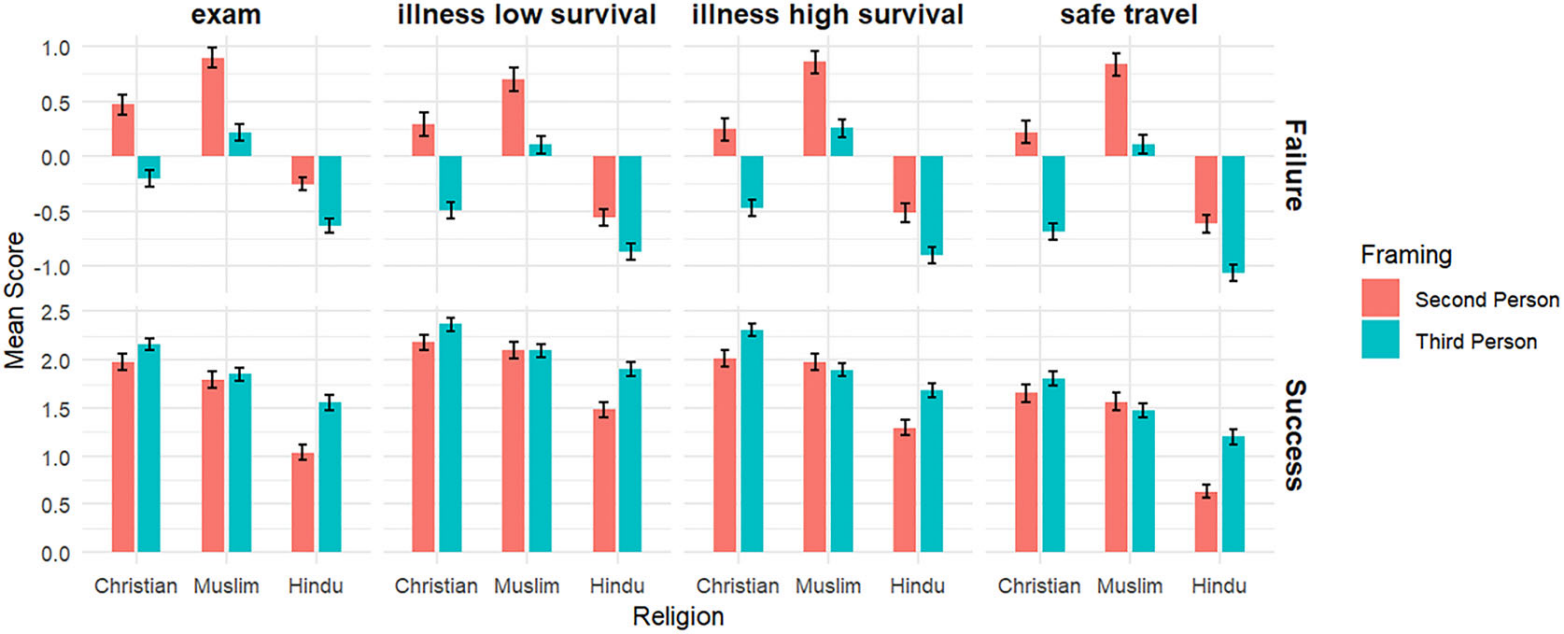
Comparisons of third vs. second person framing across different vignette scenarios and religious affiliations.

For ease of comparison, Fig. 4 juxtaposes responses across third‐ and second‐person framing, organized by religious affiliation and vignette scenario. Overall, participants tend to perceive themselves as more devout than their co‐religionists, believing that their own faith is more resistant to disconfirmation by prayer failures. Additionally, Hindu participants estimated that their co‐religionists would experience a greater boost in belief following prayer successes, suggesting that the Hindu participants expected other Hindus to exhibit a more transactional approach to divine intervention.

## 5 Discussion

### 5.1 Bayesian rationality in belief updating

Our results provide partial support for a model of belief updating that is directionally consistent with Bayesian principles in the domain of religious cognition. While belief change was generally estimated to follow a pattern consistent with Bayesian reasoning (i.e., belief increase following prayer successes and decrease following failures, especially for third‐person scenarios), the magnitude of these changes was asymmetric. Specifically, the estimated increase in belief due to prayer success was significantly larger than the decrease (if any) in belief following prayer failures. This suggests a form of evidential asymmetry in how religious believers process prayer‐related outcomes, which may be interpreted as lending support to previous research that religious beliefs tend to resist disconfirmation (Batson, 1975). This pronounced asymmetry also demonstrates that while belief change may be directionally Bayesian, it is not quantitatively so; the process may be strongly biased by motivational or social factors.

Across all three religious groups, believers estimated their own faith in God/gods to be more resilient than that of a hypothetical co‐religionist, perceiving themselves as less susceptible to belief erosion following prayer failures. However, the nature of this self‐enhancement bias varies by religious affiliation. Christians estimated that while others may experience a decline in belief in response to prayer failures, their own belief would nonetheless increase. Hindus predicted that their own belief would weaken, though to a lesser extent than that of their co‐religionists. Muslims, on the other hand, estimated that both they and their fellow Muslims would experience belief increases following prayer failures, but they expected their own increase to be of greater magnitude.

Indeed, in both our field and Prolific samples, Muslim respondents exhibit a striking pattern: Belief in God was always predicted to increase, even when prayers fail. This finding aligns with theological doctrines that frame hardships and unanswered prayers as divine tests and ultimately a blessing (Faruque & Rustom, 2023) rather than counter‐evidence against God's existence or benevolence. In contrast, non‐believers display belief updating most consistent with Bayesian principles, estimating significant decreases in belief following prayer failures (for Christian protagonists). Christians, local deity worshippers, and Hindus fall between these two extremes, with Christians and local deity worshippers exhibiting relative resistance to belief decreases and Hindus showing a more transactional approach to prayer outcomes regarding belief updating.

Interestingly, despite the doctrinal similarities between Christianity and Islam regarding divine sovereignty (Anthony Jr, 2022), Christian participants in both our field and the Prolific sample appeared more likely to follow a directionally Bayesian belief updating pattern than Muslim participants. This is likely because Christian theological traditions, particularly in many Protestant denominations, place a greater emphasis on personal responsibility and discernment in interpreting religious experiences (Ray, 1970; Stoicescu, 2021). This interpretive stance may be further shaped by the historical interplay between certain Christian traditions and cultural movements that valorize individual critical inquiry (Bennett, 2018; Ottuh, 2023). By contrast, Islamic teachings emphasize submission to divine will—indeed, the term *Islam* literally means “submission [to the will of God].” Within this framework, all events, whether perceived as positive or negative, are interpreted as part of God's greater wisdom (De Cillis, 2013), thus reducing the likelihood of belief decline even in the face of unanswered prayers.

Regarding sensitivity to prior probabilities of the prayed‐for events happening, while participants in Study 1 did not exhibit such sensitivity, in Studies 2 and 3 with much larger samples, many participants estimated greater belief increases following successful prayers for low‐probability events (i.e., recovery from a severe illness) when considering a hypothetical co‐religionist, though not themselves. This suggests that, at least in the global sample recruited from Prolific, individuals did incorporate prior probabilities into consideration when thinking about how other believers may update their beliefs in response to different prayer outcomes, consistent with Bayesian principles.

To conceptualize these two dimensions of Bayesian‐like updating: directional updating (increasing belief after successes and decreasing it after failures) represents the sign of the evidence, whereas sensitivity to priors represents the strength of the update (with more weight given to unlikely events). Observing both effects in our data suggests that participants generally recognized the valence of evidence (i.e., whether an outcome should increase or decrease belief) and, under some conditions, adjusted the magnitude of belief change based on an event's expectedness.

However, it is important to note that our findings do not demonstrate strict, quantitative Bayesian updating for two key reasons. First, the observed differences are very small, likely reflecting the broader difficulty that laypeople face when reasoning with explicit probabilistic information (Tversky & Kahneman, 1974). Second, and more critically, participants’ responses to other scenarios are inconsistent with a formal Bayesian model. The plane crash vignette is a case in point: A safe flight is an extremely high‐probability event, and from a strict Bayesian perspective, its occurrence should provide almost no new evidence to boost one's belief. Yet participants (especially Christians and Muslims) rated this prayer success as highly impactful, suggesting that the emotional salience of the outcome, rather than a formal probabilistic calculation, was likely the primary driver of the estimated belief change.

The fact that participants considered themselves as more devout (i.e., their beliefs are more resistant to prayer failures) may be due to the well documented “third‐person effect” in media and communication studies, where people believe others are more influenced by external information than they are (Davison, 1983). This effect has been observed across a wide range of domains, from advertising to political propaganda, and is thought to reflect a general self‐enhancement bias in which people protect their self‐image by attributing greater susceptibility to external forces to others rather than to themselves (Perloff, 1993). In the religious context, such a bias could translate into perceiving one's own faith as stable and resilient while assuming that co‐religionists would be more easily swayed by the outcomes of prayer. Another possibility is that individuals may view themselves as less susceptible to change because they have privileged access to their own priors. In Bayesian terms, participants may have judged that the scenarios lacked sufficient evidential weight to alter their own commitments, even if they assumed the same evidence would be more persuasive for others. Social desirability bias may also play a role—participants may have felt uncomfortable acknowledging their own susceptibility to belief revision, even when they recognized such tendencies in others (Grimm, 2010).

This, of course, does not mean that the third‐person framing is entirely reliable: participants' estimates of others' belief changes may still be influenced by their own biases, assumptions about typical religious believers, or cultural stereotypes. Additionally, third‐person judgments may reflect normative expectations rather than actual belief dynamics, as individuals might project an idealized or theoretical model of faith resilience onto others.

More generally, we would like to emphasize that our studies do not measure actual belief updating or observed behavior over time. Rather, they rely on participants’ self‐reports about how they (or others) would respond to successes and failures of petitionary prayer. These self‐reports may reflect imagined expectations or normative judgments rather than real‐world cognitive dynamics. In this sense, our findings should be interpreted as evidence of how people represent belief updating, not necessarily how they enact it in practice. Moreover, participants’ reports may also be shaped by social motives such as signaling (Sosis & Alcorta, 2003). Affirming that faith would not weaken (even in the face of unanswered prayers) can serve to demonstrate piety and group commitment. Such signaling can take different forms. Respondents might deliberately present themselves (or their co‐religionists) as steadfast believers, even if they privately expect doubts. Alternatively, they may sincerely believe their own reports, but the main purpose of those reports is to express loyalty and devotion rather than to provide an accurate account of how beliefs would actually change. In this way, statements of “resilient” faith may reveal the social function of religious commitment as much as they reflect how beliefs respond to evidence. Despite these interpretive limitations, the data remain valuable because they capture how believers think about the dynamics of faith, the norms they endorse, and the kinds of responses they consider appropriate in the face of prayer successes and failures. These perspectives offer critical insight into the representational and social dimensions of religious cognition, even if they do not track belief change directly. Future work that combines longitudinal designs with behavioral or ethnographic measures would help disentangle the extent to which these reports reflect genuine belief change, imagined expectations, or signaling functions.

Our results also indicate that non‐believers, especially from the field sample, tend to overestimate the negative impact of prayer failures on belief. However, interviews from the field sample suggest that some non‐believers do have a fairly accurate understanding of Christians’ belief dynamics:

In modern society, as long as people haven't experienced extreme hardships, their faith doesn't waver significantly. No matter what happens, they find ways to comfort themselves, telling themselves that a particular situation was just an exception and that it doesn't affect their devotion to God. My family is like this. Before something important happens, they pray, but if things don't go as they hoped, they just brush it off with an excuse to convince themselves. The next time a similar situation arises, they still turn to God for answers. So, I think unless someone experiences a truly devastating event, their faith generally won't be shaken. (Female, 20 years old)

What is particularly interesting about this response—from a non‐believer whose parents are religious—is that it not only highlights the counter‐evidence resistance of religious beliefs but also acknowledges that belief can be affected by “extreme hardships” and “truly devastating events.” These could refer to challenges of a qualitatively different magnitude than the bounded situations in our vignettes, such as famine, war, or other large‐scale catastrophes. This insight aligns with psychological theories of belief perseverance and suggests that while most religious believers are adept at rationalizing disconfirming evidence (Batson, 1975), there may be threshold events such as exceptionally severe hardships or devastation that override these rationalizations and lead to a genuine crisis of faith (Long, 2014). Future research could further investigate the conditions under which believers become more susceptible to belief revision and whether certain cultural or theological factors moderate this effect.

We acknowledge that the number of statistical comparisons across our studies is relatively large, and we did not apply formal corrections for multiple testing. This decision was guided by several considerations. First, all studies were pre‐registered and tested specific directional hypotheses derived from theory, which substantially reduces the risk of data dredging or post hoc reinterpretation (Nosek, Ebersole, DeHaven, & Mellor, 2018). Second, although corrections such as Bonferroni control family‐wise error rates, they also dramatically reduce statistical power, especially when hypotheses are theoretically motivated and focused on effect direction rather than exploration (Perneger, 1998). Third, the majority of the observed effects, especially the belief increase following prayer successes and the asymmetry relative to prayer failures, are not only statistically significant but also consistent across samples and religious traditions, providing converging support for our main claims. Nonetheless, we encourage readers to interpret marginal effects with appropriate caution and to consider the consistency of patterns across the three studies as additional support for robustness.

### 5.2 Persistence in prayer behavior

The results from both our field sample and Prolific sample largely support the hypothesis that Christians and Muslims would continue engaging in prayer despite failures. However, contrary to our expectations, local deity worshippers and Hindus also predicted that protagonists would persist in prayer even after unsuccessful outcomes, although not always to a statistically significant degree. This suggests that, while local deity worship and Hinduism may be more transactional in nature (theoretically, if a deity fails to deliver results, believers might turn to alternative supernatural agents or adjust their rituals), worshippers may still maintain religious practices due to habit, cultural expectations, or the belief that persistence will eventually elicit divine favor. A similar logic may apply to belief in human intermediaries in various religious traditions, such as faith healers. We would predict that belief in a healer's competence is transactional—analogous to the patterns we observed for local deities—because the failure can be attributed to the healer's personal limitations without challenging the believer's core faith in God.

For monotheistic believers, continued prayer despite unanswered requests may be driven by multiple psychological and cognitive mechanisms. One previously alluded key factor is the effort to reinforce faith: believers facing doubt or weakened belief may engage in more religious activities to counteract uncertainty and reaffirm their commitment, especially if they believe that the failed prayer suggests they have been “undeserving” of divine help (Hogg, Adelman, & Blagg, 2010). Additionally, the sunk cost effect may play a role. In economics, the sunk cost effect refers to the tendency to continue investing in an endeavor due to the resources already spent, even when further investment may not be rational (Arkes & Blumer, 1985). In a religious context, individuals who have devoted significant time and emotional energy to prayer and religious observance may feel compelled to persist rather than risk the psychological discomfort of abandoning their faith.

### 5.3 Implications for religious cognition and the design of religious doctrines

Our findings contribute to ongoing debates in the cognitive science of religion regarding whether religious beliefs are subject to standard evidential reasoning or if they operate under distinct epistemic rules (Boudry & Coyne, 2016b; Van Leeuwen, 2016). The observed asymmetry in belief updating, where belief increases more in response to prayer successes than it decreases (if it decreases at all) in response to failures, aligns with prior research suggesting that religious cognition incorporates strong auxiliary hypotheses that buffer core beliefs from disconfirmation (Gershman, 2019). These auxiliary hypotheses include explanations such as “God has a higher plan,” “prayer failures test faith,” or “divine intervention operates on different timescales.” However, our results also indicate that most religious followers’ beliefs are not entirely shielded from empirical counter‐evidence in the form of prayer failures, consistent with some of the recent empirical work on belief perseverance (Anglin, 2019).

It is important to acknowledge, however, that our data are open to alternative interpretations. While on the surface these practices appear instrumental—requests made to achieve specific outcomes, many scholars have emphasized the expressive, emotional, and binding roles of religious practices, such as cultivating resilience (Hamdy, 2009), displaying devotion (Henrich, 2009), or reinforcing social bonds (Whitehouse & Lanman, 2014). From these perspectives, the limited degree of belief updating in our data might simply reflect that petitionary prayers are not (or only partially) instrumental, with their persistence grounded in these non‐instrumental functions. Yet, this view is not uncontested. Other work, including our own research, has emphasized that many religious practices are deeply *instrumental* in nature, oriented toward concrete goals such as healing, protection, or success (Hong, 2022a, 2022b, 2022c; Hong et al., 2024; Hong & Henrich, 2024; Hong & Zinin, 2023). On this view, the resilience of petitionary prayer in the face of apparent disconfirmation presents a genuine puzzle: if practices are designed to deliver specific outcomes, then the buffering role of auxiliary hypotheses and doctrinal safeguards becomes central to explaining their persistence (Hong, 2025). A comprehensive review of this debate lies beyond the scope of the present paper, but we suggest it may be productive to regard petitionary prayer as at least partially instrumental. Indeed, both our survey and interview data show that successful prayers significantly boosted confidence in God/gods, indicating that their instrumental dimension is psychologically salient to practitioners.

On the theoretical front, there have been heated debates regarding the cognitive nature of religious belief, particularly the extent to which they resemble fictional imagining or factual beliefs (Levy, 2017; Szocik, 2025; Van Leeuwen, 2014). However, we must recognize that many of the features that exist extensively in religious beliefs, such as a high degree of resilience against empirical refutation, are not unique to religion. Rather, they are shared with a broader class of identity‐constituting belief systems, including deeply entrenched political ideologies, conspiratorial worldviews, pseudoscientific theories, and even certain deep‐seated moral convictions (Boudry & Coyne, 2016a).

In many of these systems, active resistance to empirical counter‐information functions as a core design feature, structuring them to minimize the impact of refutation (Boudry & Hofhuis, 2024). They incorporate epistemic defense mechanisms that allow adherents to dismiss counter‐evidence while reinforcing core commitments. In the case of petitionary prayers, religious doctrines often provide preemptive explanations for why prayers might go unanswered, thereby neutralizing potential disconfirmation. Our findings suggest that while Bayesian updating does occur, the evidential weight assigned to unsuccessful prayers is significantly diminished compared to prayer successes, reflecting these embedded doctrinal safeguards.

The unique pattern exhibited by Muslim participants—who predicted belief increases even after prayer failures—exemplifies such design feature. The Quran explicitly preempts possible doubts by invoking the omnipotence of God:

Perhaps you dislike something which is good for you and like something which is bad for you. Allah knows and you do not know. (Quran 2: 216)

Or by explicitly framing hardships and unanswered prayers as divine tests:

Do people think once they say, “We believe,” that they will be left without being put to the test? (Quran 29: 2)

Such doctrinal mechanisms provide an interpretive framework that transforms potential disconfirmation (e.g., prayer failures) into reaffirmation of faith. This aligns with cognitive theories suggesting that religious beliefs often employ self‐sealing reasoning structures (Law, 2011), wherein apparent contradictions or setbacks are reinterpreted as reinforcing rather than undermining belief, a process that scholars have noted shares important similarities with conspiracy thinking (Bezalel, 2021; Kim & Kim, 2021). Some theorists further argue that this form of belief updating, in which counter‐evidence paradoxically strengthens belief, is best understood as the product of a psychological immune system that no version of Bayesianism can fully accommodate (Mandelbaum, 2019).

From a cultural evolutionary perspective, such belief systems enjoy an advantage in that they are resistant to erosion in the face of contradictory evidence, allowing them to persist and spread across generations. By incorporating built‐in cognitive safeguards such as theological explanations for unanswered prayers, these systems reduce the likelihood of defection, ensuring high retention rates among adherents. Moreover, because these self‐sealing mechanisms mitigate doubts and reinforce group cohesion, they enhance in‐group solidarity (Van Prooijen & Van Vugt, 2018), which may contribute to their long‐term stability and cultural transmission.

While this resilience was most strikingly illustrated by our Muslim participants, who consistently predicted belief increases even after prayer failures, such protective features can be found in other monotheistic religious traditions as well. The Christian New Testament, for instance, contains passages with a similar message, urging believers to find joy in trials because “the testing of your faith produces perseverance” (James 1:2‐4, NIV). Therefore, the divergence between our Christian and Muslim samples may reflect not the absence of such doctrines in other faiths, but rather the unique theological centrality of submission in Islam, which perhaps makes the reinterpretation of setbacks as divine tests a more potent and uniform response for its adherents, especially for participants in our samples.

Building on this, future research could explore the extent to which similar epistemic defense strategies operate across different religious traditions and how they compare to analogous structures in non‐religious ideological systems, such as political or conspiracy‐driven belief systems. A promising avenue would be to investigate whether such belief systems exhibit the same tendency to treat confirming and disconfirming evidence unequally (for instance, emphasizing successes while downplaying failures), particularly in contexts where doctrinal safeguards preemptively account for disconfirmation. Understanding these parallels may offer broader insights into the cognitive mechanisms that sustain commitment to deeply held beliefs in both religious and secular domains.

## 6 Conclusion

The present studies examined the extent to which religious believers estimate belief and behavior change in response to prayer successes and failures for both themselves and co‐religionist protagonists. Through a series of vignettes and quantitative analyses, we assessed whether belief change follows Bayesian principles and whether behavioral responses to prayer outcomes differ across religious traditions. Our findings highlight an evidential asymmetry in belief updating: while most religious believers update their beliefs in response to prayer outcomes, increases in belief following prayer successes tend to be larger than decreases following prayer failures. This pattern suggests that religious belief systems are structured to absorb counter‐evidence, making them resilient to disconfirmation. At the same time, our results indicate that belief in God/gods exhibits some Bayesian features—participants showed sensitivity to prior probabilities in some contexts, and belief changes generally followed rational updating principles. This suggests that, despite its resistance to falsification, religious cognition is not entirely isolated from empirical reasoning. More empirical work is needed to carefully examine the individual, social, and cultural factors that shape religious belief systems and their evidential vulnerabilities.

## Funding information

This work was supported by The John Templeton Foundation (JTF grant ID# 61928).

## Supporting information



Supporting Information

Supporting Information

## Data Availability

All data and analyses can be found at https://github.com/kevintoy/religious_cognition.

## Notes

1 Unfortunately most of the Muslim participants declined to participate in the qualitative interviews for fear of being persecuted by the government, and many of the interviews were incomplete.

2 It is important to acknowledge, however, that both Christian and Muslim participants in the field were asked two questions regarding prayers (what they usually pray for and how they usually pray), which may have primed them to think about the potential consequences of failed prayers.
