# Supplementary material for: Evidential Vulnerability of Religious Beliefs in the Context of Petitionary Prayers
Source: Cogn Sci. 2025 Dec 28;49(12):e70163. doi: 10.1111/cogs.70163 (PMC12828874; doi:10.1111/cogs.70163)
Supplement: Supplementary file 1 — Supporting Information [file COGS-49-e70163-s001.docx]

Supplemental information for
“Evidential vulnerability of religious beliefs in the context of petitionary prayers”

## Qualtrics survey links for field participants:

Christian: <https://umac.au1.qualtrics.com/jfe/form/SV_1NvaLSUafsGm7JA>
Muslim: <https://umac.au1.qualtrics.com/jfe/form/SV_8wwrImrogB8QRpQ>
Local deity worshipper: <https://umac.au1.qualtrics.com/jfe/form/SV_7UwsJhkZnAfuv0q>
Nonbeliever: <https://umac.au1.qualtrics.com/jfe/form/SV_1TdSI0imVHtA0Z0>

## Qualtrics survey links for Prolific participants:

Christian: <https://umac.au1.qualtrics.com/jfe/form/SV_8AOWMF0ThEIYAlM>
Muslim: <https://umac.au1.qualtrics.com/jfe/form/SV_56ElYtfO2t9XGlw>
Hindu: <https://umac.au1.qualtrics.com/jfe/form/SV_aXf94j1tph8sjJ4>
Nonbeliever: <https://umac.au1.qualtrics.com/jfe/form/SV_eYidz73YFnJKLoG>

## Survey questions (in English):

Information Collection:

Gender: Male/Female/Prefer not to disclose

Age:

A. Under 18 years old

B. 18-25 years old

C. 25-40 years old

D. 40-55 years old

E. Over 55 years old

Education Level:

A. No schooling

B. Elementary school

C. Junior high school

D. High school

E. Vocational secondary school

F. Undergraduate

G. Bachelor's degree

H. Graduate degree

Qualitative Interview: Semi-structured

What is the general content of your prayers?

How do you typically pray? (At home or in church? Do you need to speak out loud? Is there a particular posture that makes prayer more effective? Are there specific times or occasions? Are certain rituals, such as baptism, or receiving blessings from a priest/communion/singing hymns, more effective?)

Over the years, has your faith in prayer and God changed? (Yes/No)

If yes, what kind of events/experiences led to this change?

(For believers only) 4. What kind of things/situations do you think would decrease a devout Christian's belief in God, or even lead to a loss of faith?

Quantitative Survey Design:

Instructions: "The following questions are presented in multiple-choice format. Please read each question carefully and select your answer as quickly and intuitively as possible."

Your level of belief in the existence of God:

1: Do not believe at all

2: Do not believe much

3: Unsure

4: Somewhat believe

5: Strongly believe

How often do you participate in religious activities such as church services, prayer, religious readings, etc.?

1: Almost never

2: 2-3 times a year

3: Once a few months

4: Once a month

5: Once a week

6: Daily or more

Xiao Ming is a devout Christian who prays and worships regularly. Before taking his college entrance exam, he prayed earnestly to God to be admitted to his desired school and also studied hard for the exam. However, when the results came out, Xiao Ming did not perform well and failed to get into the school of his choice. Do you think Xiao Ming's belief in God will change because of this?

Definitely decrease

Very likely to decrease

Might decrease

Stay the same

Might increase

Very likely to increase

Definitely increase

After repeating a year and taking the college entrance exam again, what do you think the likelihood is that Xiao Ming will pray to God?

Definitely decrease

Very likely to decrease

Might decrease

Stay the same

Might increase

Very likely to increase

Definitely increase

Xiao Qiang is also a devout Christian (who regularly prays and participates in worship). His mother was suddenly diagnosed with pancreatic cancer (which has a 90% mortality rate). He prayed earnestly to God, hoping for his mother's recovery. His mother had a strong will to live and cooperated with the treatment, but she passed away painfully a month later. Do you think Xiao Qiang's belief in God will change because of this?

definitely decrease

very likely to decrease

may decrease

stay the same

may increase

very likely to increase

definitely increase

After this, Xiao Qiang himself becomes ill in his old age, and he very much hopes to recover because he still has many worldly concerns. What do you think is the likelihood that Xiao Qiang will pray to God?

Will definitely decrease

Very likely to decrease

May decrease

Will stay the same

May increase

Very likely to increase

Will definitely increase

Xiao Hua is a devout Christian (who regularly prays and participates in worship). His mother was suddenly diagnosed with thyroid cancer (which has a 40% mortality rate). He prayed earnestly to God, asking for his mother to recover. His mother had a strong will to live and cooperated with the medical treatment, but she passed away painfully a month later. Do you think Xiao Hua's belief in God will change because of this?

Will definitely decrease

Very likely to decrease

May decrease

Stay the same

May increase

Very likely to increase

Will definitely increase

After this, Xiao Hua himself becomes ill in his old age, and he strongly hopes to recover because he still has many worldly concerns. What do you think is the likelihood that Xiao Hua will pray to God?

Will definitely decrease

Very likely to decrease

May decrease

Stay the same

May increase

Very likely to increase

Will definitely increase

Xiao Zhang is a devout Christian (who regularly prays and participates in worship). His mother was suddenly diagnosed with pancreatic cancer (which has a 90% mortality rate). He prayed earnestly to God, asking for his mother to recover. His mother had a strong will to live and cooperated with the medical treatment, and she recovered her health a month later. Do you think Xiao Zhang's belief in God will change because of this?

Will definitely decrease

Very likely to decrease

May decrease

Stay the same

May increase

Very likely to increase

Will definitely increase

After this, Xiao Zhang himself becomes ill in his old age, and he strongly hopes to recover because he still has many worldly concerns. What do you think is the likelihood that Xiao Zhang will pray to God?

Will definitely decrease

Very likely to decrease

May decrease

Stay the same

May increase

Very likely to increase

Will definitely increase

Xiao Liang is a devout Christian (who regularly prays and participates in worship). His mother was suddenly diagnosed with thyroid cancer (which has a 40% mortality rate). He prayed earnestly to God, asking for his mother to recover. His mother had a strong will to live and cooperated with the medical treatment, and she recovered her health a month later. Do you think Xiao Liang's belief in God will change because of this?

Will definitely decrease

Very likely to decrease

May decrease

Stay the same

May increase

Very likely to increase

Will definitely increase

After this, Xiao Liang himself becomes ill in his old age, and he strongly hopes to recover because he still has many worldly concerns. What do you think is the likelihood that Xiao Liang will pray to God?

Will definitely decrease

Very likely to decrease

May decrease

Stay the same

May increase

Very likely to increase

Will definitely increase

Xiao Hong is a Christian. Her father often has to travel for work, and before embarking on a long-distance flight for a business trip, Xiao Hong prayed earnestly to God for her father's safe return. Unfortunately, the plane was involved in an accident and her father tragically died. Do you think Xiao Hong's belief in God will change because of this event?

Will definitely decrease

Very likely to decrease

May decrease

Stay the same

May increase

Very likely to increase

Will definitely increase

Later, Xiao Hong’s mother also needs to take a plane for a business trip due to work. What do you think is the likelihood that Xiao Hong will pray to God?

Will definitely decrease

Very likely to decrease

May decrease

Stay the same

May increase

Very likely to increase

Will definitely increase

Xiao Bai is a Christian. His father often has to travel for work, and before going on a long-distance flight for a business trip, Xiao Bai prayed earnestly to God for his father's safe return. The flight went smoothly, and Xiao Bai's dad returned home safely after the business trip. Do you think Xiao Bai's belief in God will change because of this event?

Will definitely decrease

Very likely to decrease

May decrease

Stay the same

May increase

Very likely to increase

Will definitely increase

Afterward, Xiao Bai’s mother also needs to take a plane for a business trip due to work. What do you think is the likelihood that Xiao Bai will pray to God?

Will definitely decrease

Very likely to decrease

May decrease

Stay the same

May increase

Very likely to increase

Will definitely increase
