# Supplementary material for: Evidential Vulnerability of Religious Beliefs in the Context of Petitionary Prayers
Source: Cogn Sci. 2025 Dec 28;49(12):e70163. doi: 10.1111/cogs.70163 (PMC12828874; doi:10.1111/cogs.70163)
Supplement: Supplementary file 2 — Supporting Information [file COGS-49-e70163-s002.docx]

Supplemental information for
“Evidential vulnerability of religious beliefs in the context of petitionary prayers”
(full reports of statistical analyses)

# Belief Change After Prayer Failures (field sample, H1, One-sample *t*-test (one-tailed, alternative = "less"))

| **Group** | **Scenario** | **Mean** | **SD** | **t** | **df** | **p** | **d** |
| --- | --- | --- | --- | --- | --- | --- | --- |
| Christian | exam failure | -0.386 | 1.166 | *-2.199* | 43.000 | *0.017* | *-0.331* |
| Christian | illness low survival failure | -0.273 | 1.420 | *-1.274* | 43.000 | *0.105* | *-0.192* |
| Christian | illness high survival failure | -0.295 | 1.503 | *-1.304* | 43.000 | *0.100* | *-0.197* |
| Christian | safe travel failure | -0.674 | 1.340 | *-3.300* | 42.000 | *0.001* | *-0.503* |
| Muslim | illness high survival failure | 0.809 | 1.296 | *5.147* | 67.000 | *1.000* | *0.624* |
| Muslim | illness low survival failure | 0.714 | 1.298 | *4.603* | 69.000 | *1.000* | *0.550* |
| Muslim | safe travel failure | 0.603 | 1.211 | *4.105* | 67.000 | *1.000* | *0.498* |
| Muslim | exam failure | 0.493 | 1.080 | *3.792* | 68.000 | *1.000* | *0.456* |
| Local Deity | illness low survival failure | -0.293 | 1.327 | *-1.412* | 40.000 | *0.083* | *-0.220* |
| Local Deity | illness high survival failure | -0.125 | 1.399 | *-0.565* | 39.000 | *0.288* | *-0.089* |
| Local Deity | exam failure | -0.275 | 1.320 | *-1.317* | 39.000 | *0.098* | *-0.208* |
| Local Deity | safe travel failure | -0.525 | 1.377 | *-2.411* | 39.000 | *0.010* | *-0.381* |
| Non-believer | illness low survival failure | -1.163 | 0.986 | *-8.256* | 48.000 | *<0.001* | *-1.179* |
| Non-believer | illness high survival failure | -1.408 | 0.888 | *-11.102* | 48.000 | *<0.001* | *-1.586* |
| Non-believer | exam failure | -1.143 | 1.061 | *-7.542* | 48.000 | *<0.001* | *-1.077* |
| Non-believer | safe travel failure | -1.776 | 0.771 | *-16.121* | 48.000 | *<0.001* | *-2.303* |

# Success vs. Failure – Magnitude of Belief Change (field sample, H2, Paired-sample *t*-test (one-tailed, alternative = "greater"))

| **Group** | **Scenario** | **Mean_Success_Abs** | **SD_ Success_Abs** | **Mean_Failure_Abs** | **SD_ Failure_Abs** | **t** | **df** | **p** | **d** |
| --- | --- | --- | --- | --- | --- | --- | --- | --- | --- |
| Christian | exam success vs failure | 1.622 | 0.936 | 0.978 | 0.723 | *3.755* | 44.000 | *<0.001* | *0.560* |
| Christian | illness_low success vs failure | 2.023 | 1.000 | 1.159 | 0.861 | *5.854* | 43.000 | *<0.001* | *0.882* |
| Christian | illness_high success vs failure | 1.795 | 1.025 | 1.227 | 0.912 | *3.468* | 43.000 | *0.001* | *0.523* |
| Christian | travel success vs failure | 0.977 | 1.171 | 1.182 | 0.896 | *-1.000* | 43.000 | *0.839* | *-0.151* |
| Muslim | exam success vs failure | 1.125 | 1.310 | 0.528 | 1.074 | *4.570* | 71.000 | *<0.001* | *0.539* |
| Muslim | illness_low success vs failure | 1.250 | 1.330 | 0.750 | 1.219 | *3.855* | 71.000 | *<0.001* | *0.454* |
| Muslim | illness_high success vs failure | 1.329 | 1.334 | 0.767 | 1.242 | *4.200* | 72.000 | *<0.001* | *0.492* |
| Muslim | travel success vs failure | 1.069 | 1.314 | 0.639 | 1.166 | *3.799* | 71.000 | *<0.001* | *0.448* |
| Local Deity | exam success vs failure | 1.643 | 1.052 | 1.071 | 1.076 | *3.386* | 55.000 | *0.001* | *0.452* |
| Local Deity | illness_low success vs failure | 1.684 | 1.136 | 0.912 | 1.057 | *4.758* | 56.000 | *<0.001* | *0.630* |
| Local Deity | illness_high success vs failure | 1.571 | 1.006 | 1.125 | 0.992 | *2.739* | 55.000 | *0.004* | *0.366* |
| Local Deity | travel success vs failure | 1.429 | 1.042 | 1.089 | 0.940 | *2.242* | 55.000 | *0.015* | *0.300* |
| Non-believer | exam success vs failure | 1.545 | 0.671 | 1.364 | 0.790 | *1.073* | 21.000 | *0.148* | *0.229* |
| Non-believer | illness_low success vs failure | 2.245 | 0.879 | 1.245 | 0.879 | *5.638* | 48.000 | *<0.001* | *0.805* |
| Non-believer | illness_high success vs failure | 1.653 | 0.723 | 1.490 | 0.739 | *1.135* | 48.000 | *0.131* | *0.162* |
| Non-believer | travel success vs failure | 1.102 | 0.848 | 1.816 | 0.667 | *-4.027* | 48.000 | *1.000* | *-0.575* |

# Low vs. High Survival – Belief Change Sensitivity (field sample, H3, Paired-sample *t*-test (one-tailed, alternative = "greater"))

| **Group** | **Scenario** | **Mean_Low** | **SD_Low** | **Mean_High** | **SD_High** | **t** | **df** | **p** | **d** |
| --- | --- | --- | --- | --- | --- | --- | --- | --- | --- |
| Christian | illness success low vs high | 1.977 | 1.089 | 1.705 | 1.173 | *1.604* | 43.000 | *0.058* | *0.242* |
| Christian | illness failure low vs high | -0.289 | 1.408 | -0.267 | 1.498 | *-0.129* | 44.000 | *0.551* | *-0.019* |
| Muslim | illness success low vs high | 1.181 | 1.346 | 1.292 | 1.368 | *-1.051* | 71.000 | *0.852* | *-0.124* |
| Muslim | illness failure low vs high | 0.644 | 1.316 | 0.699 | 1.330 | *-0.469* | 72.000 | *0.680* | *-0.055* |
| Local Deity | illness success low vs high | 1.143 | 1.689 | 1.000 | 1.584 | *0.710* | 55.000 | *0.240* | *0.095* |
| Local Deity | illness failure low vs high | -0.421 | 1.336 | -0.333 | 1.456 | *-0.379* | 56.000 | *0.647* | *-0.050* |
| Non-believer | illness success low vs high | 2.163 | 1.067 | 1.571 | 0.890 | *4.795* | 48.000 | *<0.001* | *0.685* |
| Non-believer | illness failure low vs high | -1.163 | 0.986 | -1.408 | 0.888 | *1.900* | 48.000 | *0.032* | *0.271* |

# Group Comparison – Belief Decrease After Failures (field sample, H4, Independent-sample t-test (one-tailed, alternative = "greater"))

| **Comparison** | **Scenario** | **Mean_g1** | **SD_g1** | **Mean_g2** | **SD_g2** | **t** | **df** | **p** | **d** |
| --- | --- | --- | --- | --- | --- | --- | --- | --- | --- |
| Christian vs Local Deity | Exam failure | -0.400 | 1.156 | -0.632 | 1.371 | *0.925* | 100.000 | *0.179* | *0.181* |
| Christian vs Local Deity | Illness low survival failure | -0.289 | 1.408 | -0.414 | 1.325 | *0.458* | 92.000 | *0.324* | *0.092* |
| Christian vs Local Deity | Illness high survival failure | -0.267 | 1.498 | -0.333 | 1.456 | *0.226* | 93.000 | *0.411* | *0.045* |
| Christian vs Local Deity | Safe travel failure | -0.682 | 1.325 | -0.684 | 1.284 | *0.009* | 91.000 | *0.496* | *0.002* |
| Christian vs Non-believer | Exam failure | -0.400 | 1.156 | -1.143 | 1.061 | *3.237* | 89.000 | *0.001* | *0.671* |
| Christian vs Non-believer | Illness low survival failure | -0.289 | 1.408 | -1.163 | 0.986 | *3.459* | 78.000 | *<0.001* | *0.725* |
| Christian vs Non-believer | Illness high survival failure | -0.267 | 1.498 | -1.408 | 0.888 | *4.444* | 70.000 | *<0.001* | *0.937* |
| Christian vs Non-believer | Safe travel failure | -0.682 | 1.325 | -1.776 | 0.771 | *4.793* | 68.000 | *<0.001* | *1.023* |

# Behavioral Change After Prayer Failures (field sample, H5, One-sample *t*-test (one-tailed, alternative = "greater"))

| **Group** | **Scenario** | **Mean** | **SD** | **t** | **df** | **p** | **d** |
| --- | --- | --- | --- | --- | --- | --- | --- |
| Christian | exam failure | 0.659 | 0.776 | *5.635* | 43.000 | *<0.001* | *0.849* |
| Christian | illness low survival failure | 0.977 | 1.000 | *6.484* | 43.000 | *<0.001* | *0.978* |
| Christian | illness high survival failure | 0.682 | 1.029 | *4.394* | 43.000 | *<0.001* | *0.662* |
| Christian | safe travel failure | 0.535 | 1.008 | *3.479* | 42.000 | *0.001* | *0.530* |
| Muslim | illness high survival failure | 1.217 | 1.371 | *7.378* | 68.000 | *<0.001* | *0.888* |
| Muslim | illness low survival failure | 1.275 | 1.392 | *7.610* | 68.000 | *<0.001* | *0.916* |
| Muslim | safe travel failure | 1.191 | 1.406 | *6.985* | 67.000 | *<0.001* | *0.847* |
| Muslim | exam failure | 1.132 | 1.370 | *6.814* | 67.000 | *<0.001* | *0.826* |
| Local Deity | illness low survival failure | 0.450 | 1.099 | *1.831* | 19.000 | *0.041* | *0.409* |
| Local Deity | illness high survival failure | 1.059 | 1.478 | *2.954* | 16.000 | *0.005* | *0.716* |
| Local Deity | exam failure | 0.375 | 1.025 | *1.464* | 15.000 | *0.082* | *0.366* |
| Local Deity | safe travel failure | 0.833 | 1.383 | *2.557* | 17.000 | *0.010* | *0.603* |
| Non-believer | illness low survival failure | -0.143 | 1.208 | *-0.828* | 48.000 | *0.794* | *-0.118* |
| Non-believer | illness high survival failure | -0.286 | 1.369 | *-1.461* | 48.000 | *0.925* | *-0.209* |
| Non-believer | exam failure | -0.327 | 1.505 | *-1.518* | 48.000 | *0.932* | *-0.217* |
| Non-believer | safe travel failure | -0.429 | 1.620 | *-1.852* | 48.000 | *0.965* | *-0.265* |

# Belief Change After Prayer Failures (third-person Global Sample, H1, One-sample *t*-test (one-tailed, alternative = "less"))

| **Group** | **Scenario** | **Mean** | **SD** | **t** | **df** | **p** | **d** |
| --- | --- | --- | --- | --- | --- | --- | --- |
| Christian | exam failure | -0.206 | 1.278 | *-2.818* | 305.000 | *0.003* | *-0.161* |
| Christian | illness low survival failure | -0.490 | 1.339 | *-6.406* | 305.000 | *<0.001* | *-0.366* |
| Christian | illness high survival failure | -0.474 | 1.363 | *-6.083* | 305.000 | *<0.001* | *-0.348* |
| Christian | safe travel failure | -0.691 | 1.321 | *-9.163* | 306.000 | *<0.001* | *-0.523* |
| Muslim | exam failure | 0.215 | 1.333 | *2.846* | 311.000 | *0.998* | *0.161* |
| Muslim | illness low survival failure | 0.106 | 1.447 | *1.291* | 311.000 | *0.901* | *0.073* |
| Muslim | illness high survival failure | 0.256 | 1.438 | *3.149* | 311.000 | *0.999* | *0.178* |
| Muslim | safe travel failure | 0.107 | 1.501 | *1.251* | 308.000 | *0.894* | *0.071* |
| Hindu | exam failure | -0.635 | 0.921 | *-10.202* | 218.000 | *<0.001* | *-0.689* |
| Hindu | illness low survival failure | -0.872 | 1.080 | *-11.947* | 218.000 | *<0.001* | *-0.807* |
| Hindu | illness high survival failure | -0.905 | 1.133 | *-11.844* | 219.000 | *<0.001* | *-0.799* |
| Hindu | safe travel failure | -1.064 | 1.156 | *-13.625* | 218.000 | *<0.001* | *-0.921* |
| Non-believer | exam failure | -0.640 | 0.811 | *-7.896* | 99.000 | *<0.001* | *-0.790* |
| Non-believer | illness low survival failure | -0.880 | 0.956 | *-9.201* | 99.000 | *<0.001* | *-0.920* |
| Non-believer | illness high survival failure | -1.180 | 0.989 | *-11.936* | 99.000 | *<0.001* | *-1.194* |
| Non-believer | safe travel failure | -1.110 | 1.004 | *-11.056* | 99.000 | *<0.001* | *-1.106* |

# Success vs. Failure – Magnitude of Belief Change (third-person Global Sample, H2, Paired-sample *t*-test (one-tailed, alternative = "greater"))

| **Group** | **Scenario** | **Mean_ Success_Abs** | **SD_ Success_ Abs** | **Mean_ Failure_ Abs** | **SD_ Failure_Abs** | **t** | **df** | **p** | **d** |
| --- | --- | --- | --- | --- | --- | --- | --- | --- | --- |
| Christian | exam success vs failure | 2.180 | 0.994 | 0.912 | 0.917 | *18.179* | 305.000 | *<0.001* | *1.039* |
| Christian | illness low success vs failure | 2.490 | 0.844 | 1.030 | 0.976 | *21.289* | 303.000 | *<0.001* | *1.221* |
| Christian | illness high success vs failure | 2.376 | 0.905 | 1.082 | 0.953 | *18.524* | 305.000 | *<0.001* | *1.059* |
| Christian | travel success vs failure | 1.840 | 1.236 | 1.160 | 0.934 | *8.629* | 306.000 | *<0.001* | *0.492* |
| Muslim | exam success vs failure | 1.920 | 1.086 | 0.913 | 0.993 | *14.746* | 311.000 | *<0.001* | *0.835* |
| Muslim | illness low success vs failure | 2.189 | 1.020 | 1.003 | 1.047 | *16.600* | 311.000 | *<0.001* | *0.940* |
| Muslim | illness high success vs failure | 1.987 | 1.008 | 1.032 | 1.033 | *14.494* | 311.000 | *<0.001* | *0.821* |
| Muslim | travel success vs failure | 1.524 | 1.245 | 1.104 | 1.020 | *5.808* | 308.000 | *<0.001* | *0.330* |
| Hindu | exam success vs failure | 1.658 | 1.026 | 0.799 | 0.782 | *10.411* | 218.000 | *<0.001* | *0.703* |
| Hindu | illness low success vs failure | 1.972 | 1.007 | 1.037 | 0.925 | *10.801* | 217.000 | *<0.001* | *0.732* |
| Hindu | illness high success vs failure | 1.777 | 0.937 | 1.141 | 0.893 | *7.947* | 219.000 | *<0.001* | *0.536* |
| Hindu | travel success vs failure | 1.247 | 1.089 | 1.274 | 0.918 | *-0.293* | 218.000 | *0.615* | *-0.020* |
| Non-believer | exam success vs failure | 1.000 | 0.816 | 0.700 | 0.759 | *2.649* | 99.000 | *0.005* | *0.265* |
| Non-believer | illness low success vs failure | 2.070 | 0.956 | 0.980 | 0.853 | *8.850* | 99.000 | *<0.001* | *0.885* |
| Non-believer | illness high success vs failure | 1.530 | 0.915 | 1.300 | 0.823 | *2.025* | 99.000 | *0.023* | *0.203* |
| Non-believer | travel success vs failure | 0.530 | 0.758 | 1.250 | 0.821 | *-6.888* | 99.000 | *1.000* | *-0.689* |

# Low vs. High Survival – Belief Change Sensitivity (third-person Global Sample, H3, Paired-sample *t*-test (one-tailed, alternative = "greater"))

| **Group** | **Scenario** | **Mean_Low** | **SD_Low** | **Mean_High** | **SD_High** | **t** | **df** | **p** | **d** |
| --- | --- | --- | --- | --- | --- | --- | --- | --- | --- |
| Christian | illness success low vs high | 2.365 | 1.150 | 2.299 | 1.077 | *1.069* | 303.000 | *0.143* | *0.061* |
| Christian | illness failure low vs high | -0.490 | 1.339 | -0.474 | 1.363 | *-0.236* | 305.000 | *0.593* | *-0.013* |
| Muslim | illness success low vs high | 2.099 | 1.195 | 1.897 | 1.169 | *3.314* | 311.000 | *0.001* | *0.188* |
| Muslim | illness failure low vs high | 0.106 | 1.447 | 0.256 | 1.438 | *-2.046* | 311.000 | *0.979* | *-0.116* |
| Hindu | illness success low vs high | 1.904 | 1.139 | 1.680 | 1.091 | *2.861* | 218.000 | *0.002* | *0.193* |
| Hindu | illness failure low vs high | -0.872 | 1.080 | -0.922 | 1.104 | *0.696* | 218.000 | *0.243* | *0.047* |
| Non-believer | illness success low vs high | 2.070 | 0.956 | 1.410 | 1.093 | *6.983* | 99.000 | *<0.001* | *0.698* |
| Non-believer | illness failure low vs high | -0.880 | 0.956 | -1.180 | 0.989 | *3.129* | 99.000 | *0.001* | *0.313* |

# Group Comparison – Belief Decrease After Failures (third-person Global Sample, H4, Independent-sample *t*-test (one-tailed, alternative = "greater"))

| **Comparison** | **Scenario** | **Mean_g1** | **SD_g1** | **Mean_g2** | **SD_g2** | **t** | **df** | **p** | **d** |
| --- | --- | --- | --- | --- | --- | --- | --- | --- | --- |
| Christian vs Hindu | exam failure | -0.206 | 1.278 | -0.635 | 0.921 | *4.469* | 523.000 | *<0.001* | *0.375* |
| Christian vs Hindu | illness low survival failure | -0.490 | 1.339 | -0.872 | 1.080 | *3.612* | 515.000 | *<0.001* | *0.309* |
| Christian vs Hindu | illness high survival failure | -0.474 | 1.363 | -0.905 | 1.133 | *3.948* | 513.000 | *<0.001* | *0.339* |
| Christian vs Hindu | safe travel failure | -0.691 | 1.321 | -1.064 | 1.156 | *3.440* | 503.000 | *<0.001* | *0.298* |
| Christian vs Non-believer | exam failure | -0.206 | 1.278 | -0.640 | 0.811 | *3.979* | 268.000 | *<0.001* | *0.368* |
| Christian vs Non-believer | illness low survival failure | -0.490 | 1.339 | -0.880 | 0.956 | *3.182* | 235.000 | *<0.001* | *0.310* |
| Christian vs Non-believer | illness high survival failure | -0.474 | 1.363 | -1.180 | 0.989 | *5.610* | 231.000 | *<0.001* | *0.551* |
| Christian vs Non-believer | safe travel failure | -0.691 | 1.321 | -1.110 | 1.004 | *3.341* | 219.000 | *<0.001* | *0.335* |

# Behavioral Change After Prayer Failures (third-person Global Sample, H5, One-sample *t*-test (one-tailed, alternative = "greater"))

| **Group** | **Scenario** | **Mean** | **SD** | **t** | **df** | **p** | **d** |
| --- | --- | --- | --- | --- | --- | --- | --- |
| Christian | exam failure | 0.775 | 1.477 | *9.197* | 306.000 | *<0.001* | *0.525* |
| Christian | illness low survival failure | 0.550 | 1.599 | *6.034* | 306.000 | *<0.001* | *0.344* |
| Christian | illness high survival failure | 0.570 | 1.485 | *6.724* | 306.000 | *<0.001* | *0.384* |
| Christian | safe travel failure | 0.664 | 1.808 | *6.439* | 306.000 | *<0.001* | *0.367* |
| Muslim | exam failure | 1.087 | 1.415 | *13.564* | 311.000 | *<0.001* | *0.768* |
| Muslim | illness low survival failure | 1.288 | 1.444 | *15.766* | 311.000 | *<0.001* | *0.893* |
| Muslim | illness high survival failure | 1.083 | 1.528 | *12.526* | 311.000 | *<0.001* | *0.709* |
| Muslim | safe travel failure | 1.148 | 1.678 | *12.062* | 310.000 | *<0.001* | *0.684* |
| Hindu | exam failure | 0.333 | 1.261 | *3.912* | 218.000 | *<0.001* | *0.264* |
| Hindu | illness low survival failure | 0.251 | 1.400 | *2.655* | 218.000 | *0.004* | *0.179* |
| Hindu | illness high survival failure | 0.127 | 1.459 | *1.294* | 219.000 | *0.099* | *0.087* |
| Hindu | safe travel failure | 0.165 | 1.618 | *1.507* | 217.000 | *0.067* | *0.102* |
| Non-believer | exam failure | -0.010 | 1.133 | *-0.088* | 99.000 | *0.535* | *-0.009* |
| Non-believer | illness low survival failure | 0.080 | 1.475 | *0.542* | 99.000 | *0.294* | *0.054* |
| Non-believer | illness high survival failure | -0.130 | 1.346 | *-0.966* | 99.000 | *0.832* | *-0.097* |
| Non-believer | safe travel failure | 0.150 | 1.666 | *0.900* | 99.000 | *0.185* | *0.090* |

# Belief Change After Prayer Failures (second-person Global Sample, H1, One-sample *t*-test (one-tailed, alternative = "less"))

| **Group** | **Scenario** | **Mean** | **SD** | **t** | **df** | **p** | **d** |
| --- | --- | --- | --- | --- | --- | --- | --- |
| Christian | exam failure | 0.473 | 1.304 | *5.194* | 204.000 | *1.000* | *0.363* |
| Christian | illness low survival failure | 0.293 | 1.499 | *2.795* | 204.000 | *0.997* | *0.195* |
| Christian | illness high survival failure | 0.244 | 1.472 | *2.373* | 204.000 | *0.991* | *0.166* |
| Christian | safe travel failure | 0.220 | 1.480 | *2.123* | 204.000 | *0.983* | *0.148* |
| Muslim | exam failure | 0.899 | 1.391 | *9.518* | 216.000 | *1.000* | *0.646* |
| Muslim | illness low survival failure | 0.702 | 1.599 | *6.442* | 214.000 | *1.000* | *0.439* |
| Muslim | illness high survival failure | 0.860 | 1.504 | *8.391* | 214.000 | *1.000* | *0.572* |
| Muslim | safe travel failure | 0.838 | 1.493 | *8.248* | 215.000 | *1.000* | *0.561* |
| Hindu | exam failure | -0.253 | 0.905 | *-4.124* | 216.000 | *<0.001* | *-0.280* |
| Hindu | illness low survival failure | -0.560 | 1.160 | *-7.098* | 215.000 | *<0.001* | *-0.483* |
| Hindu | illness high survival failure | -0.516 | 1.273 | *-5.971* | 216.000 | *<0.001* | *-0.405* |
| Hindu | safe travel failure | -0.618 | 1.181 | *-7.703* | 216.000 | *<0.001* | *-0.523* |

# Success vs. Failure – Magnitude of Belief Change (Second-Person Global Sample, H2, Paired-sample *t*-test (one-tailed, alternative = "greater"))

| **Group** | **Scenario** | **Mean_ Success_Abs** | **SD_ Success_Abs** | **Mean_ Failure_Abs** | **SD_ Failure_Abs** | **t** | **df** | **p** | **d** |
| --- | --- | --- | --- | --- | --- | --- | --- | --- | --- |
| Christian | exam success vs failure | 1.980 | 1.196 | 0.834 | 1.108 | *12.835* | 204.000 | *<0.001* | *0.896* |
| Christian | illness low success vs failure | 2.205 | 1.101 | 0.976 | 1.173 | *13.319* | 204.000 | *<0.001* | *0.930* |
| Christian | illness high success vs failure | 2.039 | 1.178 | 0.931 | 1.160 | *11.888* | 203.000 | *<0.001* | *0.832* |
| Christian | travel success vs failure | 1.683 | 1.307 | 0.980 | 1.129 | *6.294* | 204.000 | *<0.001* | *0.440* |
| Muslim | exam success vs failure | 1.824 | 1.241 | 1.042 | 1.292 | *9.564* | 215.000 | *<0.001* | *0.651* |
| Muslim | illness low success vs failure | 2.130 | 1.161 | 1.158 | 1.305 | *10.615* | 214.000 | *<0.001* | *0.724* |
| Muslim | illness high success vs failure | 2.033 | 1.175 | 1.146 | 1.293 | *9.631* | 212.000 | *<0.001* | *0.660* |
| Muslim | travel success vs failure | 1.593 | 1.349 | 1.136 | 1.291 | *5.750* | 213.000 | *<0.001* | *0.393* |
| Hindu | exam success vs failure | 1.093 | 1.079 | 0.486 | 0.807 | *7.428* | 215.000 | *<0.001* | *0.505* |
| Hindu | illness low success vs failure | 1.546 | 1.069 | 0.829 | 0.985 | *8.439* | 215.000 | *<0.001* | *0.574* |
| Hindu | illness high success vs failure | 1.387 | 1.017 | 0.922 | 1.018 | *5.687* | 216.000 | *<0.001* | *0.386* |
| Hindu | travel success vs failure | 0.705 | 0.979 | 0.885 | 0.996 | *-2.129* | 216.000 | *0.983* | *-0.145* |

# Low vs. High Survival – Belief Change Sensitivity (Second-Person Global Sample, H3, Paired-sample *t*-test (one-tailed, alternative = "greater"))

| **Group** | **Scenario** | **Mean_Low** | **SD_Low** | **Mean_High** | **SD_High** | **t** | **df** | **p** | **d** |
| --- | --- | --- | --- | --- | --- | --- | --- | --- | --- |
| Christian | illness success low vs high | 2.186 | 1.142 | 2.010 | 1.228 | *2.195* | 203.000 | *0.015* | *0.154* |
| Christian | illness failure low vs high | 0.293 | 1.499 | 0.244 | 1.472 | *0.658* | 204.000 | *0.255* | *0.046* |
| Muslim | illness success low vs high | 2.103 | 1.213 | 1.977 | 1.268 | *2.160* | 212.000 | *0.016* | *0.148* |
| Muslim | illness failure low vs high | 0.702 | 1.599 | 0.860 | 1.504 | *-1.906* | 214.000 | *0.971* | *-0.130* |
| Hindu | illness success low vs high | 1.484 | 1.155 | 1.295 | 1.133 | *2.690* | 216.000 | *0.004* | *0.183* |
| Hindu | illness failure low vs high | -0.560 | 1.160 | -0.523 | 1.272 | *-0.583* | 215.000 | *0.720* | *-0.040* |

# Group Comparison – Belief Decrease After Failures (Second-Person Global Sample, H4, Independent-sample *t*-test (one-tailed, alternative = "greater"))

| **Comparison** | **Scenario** | **Mean_ Christian** | **SD_ Christian** | **Mean_ Hindu** | **SD_ Hindu** | **t** | **df** | **p** | **d** |
| --- | --- | --- | --- | --- | --- | --- | --- | --- | --- |
| Christian vs Hindu | exam failure | 0.473 | 1.304 | -0.253 | 0.905 | *6.612* | 361.272 | *<0.001* | *0.650* |
| Christian vs Hindu | illness low survival failure | 0.293 | 1.499 | -0.560 | 1.160 | *6.505* | 384.027 | *<0.001* | *0.638* |
| Christian vs Hindu | illness high survival failure | 0.244 | 1.472 | -0.516 | 1.273 | *5.659* | 403.822 | *<0.001* | *0.553* |
| Christian vs Hindu | safe travel failure | 0.220 | 1.480 | -0.618 | 1.181 | *6.397* | 389.911 | *<0.001* | *0.627* |

# Behavioral Change After Prayer Failures (Second-Person Global Sample, H5, One-sample *t*-test (one-tailed, alternative = "greater"))

| **Group** | **Scenario** | **Mean** | **SD** | **t** | **df** | **p** | **d** |
| --- | --- | --- | --- | --- | --- | --- | --- |
| Christian | exam failure | 1.745 | 1.329 | *18.750* | 203.000 | *<0.001* | *1.313* |
| Christian | illness low survival failure | 1.595 | 1.342 | *17.018* | 204.000 | *<0.001* | *1.189* |
| Christian | illness high survival failure | 1.488 | 1.399 | *15.228* | 204.000 | *<0.001* | *1.064* |
| Christian | safe travel failure | 1.683 | 1.449 | *16.628* | 204.000 | *<0.001* | *1.161* |
| Muslim | exam failure | 1.874 | 1.321 | *20.801* | 214.000 | *<0.001* | *1.419* |
| Muslim | illness low survival failure | 1.825 | 1.336 | *20.126* | 216.000 | *<0.001* | *1.366* |
| Muslim | illness high survival failure | 1.847 | 1.322 | *20.530* | 215.000 | *<0.001* | *1.397* |
| Muslim | safe travel failure | 1.889 | 1.332 | *20.845* | 215.000 | *<0.001* | *1.418* |
| Hindu | exam failure | 0.429 | 1.249 | *5.053* | 216.000 | *<0.001* | *0.343* |
| Hindu | illness low survival failure | 0.286 | 1.378 | *3.054* | 216.000 | *0.001* | *0.207* |
| Hindu | illness high survival failure | 0.147 | 1.356 | *1.602* | 216.000 | *0.055* | *0.109* |
| Hindu | safe travel failure | 0.267 | 1.498 | *2.629* | 216.000 | *0.005* | *0.178* |

# Perspective Comparison – Third-Person vs. Second-Person Scenarios (Global Sample, H6, Independent-sample *t*-test (two-tailed))

| **group** | **Scenario** | **Mean_Third** | **Mean_Second** | **t** | **df** | **p** | **d** |
| --- | --- | --- | --- | --- | --- | --- | --- |
| Christian | exam failure (behavior) | 0.775 | 1.745 | *7.724* | 465.000 | *<0.001* | *0.683* |
| Christian | exam failure (belief) | -0.206 | 0.473 | *5.815* | 431.000 | *<0.001* | *0.527* |
| Christian | exam success (behavior) | 2.143 | 2.195 | *0.521* | 437.000 | *0.603* | *0.047* |
| Christian | exam success (belief) | 2.163 | 1.980 | *-1.782* | 393.000 | *0.075* | *-0.165* |
| Christian | illness high survival failure (behavior) | 0.570 | 1.488 | *7.095* | 455.000 | *<0.001* | *0.632* |
| Christian | illness high survival failure (belief) | -0.474 | 0.244 | *5.566* | 414.000 | *<0.001* | *0.510* |
| Christian | illness high survival success (behavior) | 2.173 | 2.127 | *-0.462* | 452.000 | *0.644* | *-0.041* |
| Christian | illness high survival success (belief) | 2.304 | 2.010 | *-2.783* | 395.000 | *0.006* | *-0.258* |
| Christian | illness low survival failure (behavior) | 0.550 | 1.595 | *7.986* | 484.000 | *<0.001* | *0.696* |
| Christian | illness low survival failure (belief) | -0.490 | 0.293 | *6.037* | 403.000 | *<0.001* | *0.557* |
| Christian | illness low survival success (behavior) | 2.208 | 2.127 | *-0.813* | 453.000 | *0.417* | *-0.073* |
| Christian | illness low survival success (belief) | 2.365 | 2.185 | *-1.739* | 440.000 | *0.083* | *-0.157* |
| Christian | safe travel failure (behavior) | 0.664 | 1.683 | *7.046* | 493.000 | *<0.001* | *0.608* |
| Christian | safe travel failure (belief) | -0.691 | 0.220 | *7.113* | 403.000 | *<0.001* | *0.656* |
| Christian | safe travel success (behavior) | 2.010 | 2.005 | *-0.046* | 443.000 | *0.964* | *-0.004* |
| Christian | safe travel success (belief) | 1.801 | 1.654 | *-1.237* | 426.000 | *0.217* | *-0.112* |
| Hindu | exam failure (behavior) | 0.333 | 0.429 | *0.792* | 434.000 | *0.429* | *0.076* |
| Hindu | exam failure (belief) | -0.635 | -0.253 | *4.360* | 434.000 | *<0.001* | *0.418* |
| Hindu | exam success (behavior) | 1.507 | 1.120 | *-3.528* | 433.000 | *<0.001* | *-0.338* |
| Hindu | exam success (belief) | 1.557 | 1.037 | *-4.704* | 433.000 | *<0.001* | *-0.451* |
| Hindu | illness high survival failure (behavior) | 0.127 | 0.147 | *0.150* | 433.000 | *0.881* | *0.014* |
| Hindu | illness high survival failure (belief) | -0.905 | -0.516 | *3.368* | 428.000 | *0.001* | *0.322* |
| Hindu | illness high survival success (behavior) | 1.709 | 1.041 | *-6.397* | 434.000 | *<0.001* | *-0.612* |
| Hindu | illness high survival success (belief) | 1.686 | 1.295 | *-3.676* | 434.000 | *<0.001* | *-0.352* |
| Hindu | illness low survival failure (behavior) | 0.251 | 0.286 | *0.260* | 434.000 | *0.795* | *0.025* |
| Hindu | illness low survival failure (belief) | -0.872 | -0.560 | *2.902* | 430.000 | *0.004* | *0.278* |
| Hindu | illness low survival success (behavior) | 1.877 | 1.221 | *-6.018* | 433.000 | *<0.001* | *-0.577* |
| Hindu | illness low survival success (belief) | 1.904 | 1.484 | *-3.824* | 434.000 | *<0.001* | *-0.366* |
| Hindu | safe travel failure (behavior) | 0.165 | 0.267 | *0.683* | 431.000 | *0.495* | *0.066* |
| Hindu | safe travel failure (belief) | -1.064 | -0.618 | *3.989* | 434.000 | *<0.001* | *0.382* |
| Hindu | safe travel success (behavior) | 1.368 | 0.779 | *-5.512* | 426.000 | *<0.001* | *-0.527* |
| Hindu | safe travel success (belief) | 1.200 | 0.631 | *-5.442* | 431.000 | *<0.001* | *-0.520* |
| Muslim | exam failure (behavior) | 1.087 | 1.874 | *6.535* | 480.000 | *<0.001* | *0.572* |
| Muslim | exam failure (belief) | 0.215 | 0.899 | *5.658* | 452.000 | *<0.001* | *0.504* |
| Muslim | exam success (behavior) | 1.853 | 1.963 | *1.026* | 464.000 | *0.305* | *0.091* |
| Muslim | exam success (belief) | 1.849 | 1.796 | *-0.480* | 443.000 | *0.632* | *-0.043* |
| Muslim | illness high survival failure (behavior) | 1.083 | 1.847 | *6.121* | 500.000 | *<0.001* | *0.528* |
| Muslim | illness high survival failure (belief) | 0.256 | 0.860 | *4.613* | 447.000 | *<0.001* | *0.412* |
| Muslim | illness high survival success (behavior) | 2.000 | 2.069 | *0.701* | 442.000 | *0.483* | *0.063* |
| Muslim | illness high survival success (belief) | 1.897 | 1.977 | *0.724* | 430.000 | *0.469* | *0.065* |
| Muslim | illness low survival failure (behavior) | 1.288 | 1.825 | *4.395* | 487.000 | *<0.001* | *0.383* |
| Muslim | illness low survival failure (belief) | 0.106 | 0.702 | *4.374* | 430.000 | *<0.001* | *0.395* |
| Muslim | illness low survival success (behavior) | 2.103 | 2.166 | *0.659* | 455.000 | *0.510* | *0.059* |
| Muslim | illness low survival success (belief) | 2.099 | 2.102 | *0.028* | 456.000 | *0.978* | *0.002* |
| Muslim | safe travel failure (behavior) | 1.148 | 1.889 | *5.639* | 516.000 | *<0.001* | *0.479* |
| Muslim | safe travel failure (belief) | 0.107 | 0.838 | *5.510* | 464.000 | *<0.001* | *0.488* |
| Muslim | safe travel success (behavior) | 1.439 | 1.788 | *3.037* | 467.000 | *0.003* | *0.268* |
| Muslim | safe travel success (belief) | 1.478 | 1.565 | *0.732* | 441.000 | *0.464* | *0.066* |
